# A Precious‐Metal‐Free Hybrid Electrolyzer for Alcohol Oxidation Coupled to CO_2_‐to‐Syngas Conversion

**DOI:** 10.1002/anie.202002680

**Published:** 2020-05-18

**Authors:** Mark A. Bajada, Souvik Roy, Julien Warnan, Kaltum Abdiaziz, Andreas Wagner, Maxie M. Roessler, Erwin Reisner

**Affiliations:** ^1^ Department of Chemistry University of Cambridge Cambridge CB2 1EW UK; ^2^ Department of Chemistry Imperial College London Molecular Sciences Research Hub White City Campus London W12 0BZ UK; ^3^ School of Biological and Chemical Sciences and Materials Research Institute Queen Mary University of London London E1 4NS UK

**Keywords:** alcohols, carbon dioxide, catalyst immobilization, electrocatalysis, energy conversion

## Abstract

Electrolyzers combining CO_2_ reduction (CO_2_R) with organic substrate oxidation can produce fuel and chemical feedstocks with a relatively low energy requirement when compared to systems that source electrons from water oxidation. Here, we report an anodic hybrid assembly based on a (2,2,6,6‐tetramethylpiperidin‐1‐yl)oxyl (TEMPO) electrocatalyst modified with a silatrane‐anchor (**STEMPO**), which is covalently immobilized on a mesoporous indium tin oxide (*meso*ITO) scaffold for efficient alcohol oxidation (AlcOx). This molecular anode was subsequently combined with a cathode consisting of a polymeric cobalt phthalocyanine on carbon nanotubes to construct a hybrid, precious‐metal‐free coupled AlcOx–CO_2_R electrolyzer. After three‐hour electrolysis, glycerol is selectively oxidized to glyceraldehyde with a turnover number (TON) of ≈1000 and Faradaic efficiency (FE) of 83 %. The cathode generated a stoichiometric amount of syngas with a CO:H_2_ ratio of 1.25±0.25 and an overall cobalt‐based TON of 894 with a FE of 82 %. This prototype device inspires the design and implementation of nonconventional strategies for coupling CO_2_R to less energy demanding, and value‐added, oxidative chemistry.

## Graphical abstract

**Inspirational device**: A molecularly engineered electrolyzer was constructed for the selective oxidation of glycerol to glyceraldehyde, with a turnover number (TON) of ≈1000 and a Faradaic efficiency (FE) of 83 %, coupled with aqueous CO_2_ reduction to generate a stoichiometric amount of syngas, with a CO:H_2_ ratio of 1.25±0.25, an overall TON of 894 and FE of 82 %.

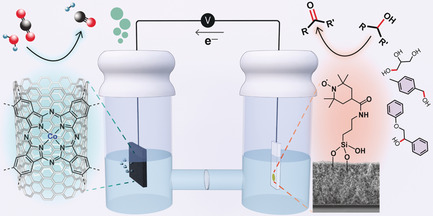

## Introduction

The electrosynthesis of fuels is being pursued as a potential solution to intermittent electricity production via renewable wind and solar technologies.^1^ Conventional fuel‐generating electrolyzers couple the oxygen evolution reaction (OER) at the anode to the hydrogen evolution reaction (HER) or CO_2_R at the cathode.^2^, ^3^ However, the kinetic hurdles of the anodic four‐electron process and consequently large overpotential for the OER, tied to the limited commercial value of O_2_, are spurring interest in employing more synthetically useful and facile organic oxidative reactions.^4^, ^5^, ^6^, ^7^, ^8^, ^9^, ^10^, ^11^

Recent technoeconomic studies have shown that ≈90 % of the overall energy requirements for current commercial approaches in CO_2_ electrolysis stem from the OER, and that lower cell potentials for fuel‐generating reductive chemistry can be achieved by substituting the OER for AlcOx.^12^ In particular, by combining theory and experiment, it was shown that glycerol, a biomass‐derived platform chemical and a by‐product from the production of biodiesel and soap,^13^, ^14^ is an attractive candidate that can greatly improve the economics of the overall redox process.

Reports on dual AlcOx–CO_2_R electrolyzers suffer from two main drawbacks to date.^7^, ^9^ Firstly, precious metal‐containing components are employed in the electrolysis cells. Secondly, homogeneous catalysts and mediators are required in excess in the electrolyte solution, which complicates post‐reaction processing of the liquid products. The use of dissolved catalysts presents an additional challenge during electrosynthesis, because only a tiny fraction of the catalyst (positioned in the Helmholtz layer) can turnover, while the rest remains inactive in the bulk solution.

**Figure 1:**
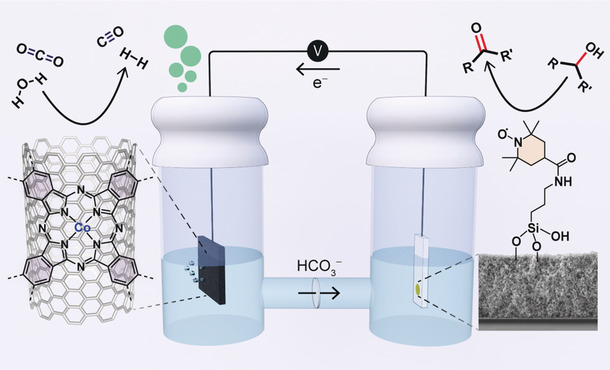
Coupled AlcOx–CO_2_R electrolyzer, featuring a *meso*ITO|**STEMPO** anode (right‐hand compartment) and a CP|CNT‐**CoPPc** cathode (left‐hand compartment). An SEM cross‐section image of the *meso*ITO electrode is shown on the right (film thickness ≈4.5 μm).

Here, we consider glycerol as a commercially viable resource to develop a robust and precious‐metal‐free anodic assembly for coupling with the cathodic CO_2_R reaction. We modified TEMPO, an efficient, non‐toxic, molecular (electro)catalyst that can oxidize a wide range of alcohol substrates under mild conditions,^15^, ^16^ with a silatrane anchor (giving **STEMPO**), for robust immobilization on a *meso*ITO scaffold (Figure 1). The porous electrode enables high catalyst loading, while the immobilization procedure permits direct electronic communication between the electrode and the molecular species, leading to constant catalytic turnover, easier product isolation and catalyst recyclability. The performance of the *meso*ITO|**STEMPO** assembly was assessed under different conditions with a range of alcohols.

We then coupled the anode to a hybrid CO_2_R cathode, featuring a cobalt phthalocyanine (**CoPc**) based electrocatalyst. **CoPc** was polymerized onto carbon nanotubes (CNTs) to form a CNT‐polymeric **CoPc** composite (CNT‐**CoPPc**; where **CoPPc** denotes polymeric cobalt phthalocyanine), which was then deposited onto porous carbon paper (CP, cathode assembly henceforth denoted as CP|CNT‐**CoPPc**)^17^ (Figure 1). The AlcOx–CO_2_R electrolyzer oxidizes glycerol to glyceraldehyde with good catalytic performance and high selectivity, and generates a stoichiometric amount of syngas at the cathode.

## Results and Discussion

Metal oxide (MO_*x*_) electrodes present a suitable platform for catalyst immobilization as they offer affinity for a variety of anchoring units, and the possibility to morphologically tune the surface to enhance the loading of molecular components.^18^, ^19^ Metal oxides can exhibit different electronic properties, as demonstrated by the metallic behavior of ITO and the semiconductive properties of TiO_2_, thus offering a versatile electroactive platform to combine with surface‐anchored molecular catalysts.^20^, ^21^ Several mechanistic studies have highlighted the effect of pH on the TEMPO catalytic cycle, with enhanced oxidation rates observed under more basic conditions.^22^, ^23^, ^24^ This stringent criterion implies that some of the more commonly used anchoring groups compatible with MO_*x*_ scaffolds, such as carboxylic acids and phosphonic acids (pH stability <4 and 7, respectively),^25^ may not be suitable for TEMPO immobilization on an ITO electrode. We therefore designed **STEMPO**, which contains a caged silatrane unit to improve binding to the MO_*x*_. The silatrane moiety can hydrolyze on the MO_*x*_ surface to form strong siloxane bonds, which provide an increased anchor stability under more alkaline conditions (Figure 1).^26^, ^27^

**STEMPO** was synthesized in good yield by reacting the acyl chloride of 4‐carboxy‐TEMPO with 3‐aminopropylsilatrane. Full synthetic and characterization details (high‐resolution mass spectrometry, infrared spectroscopy (Figure S1) and elemental analysis) are provided in the Supporting Information.

The *meso*ITO|**STEMPO** anode was assembled by incubating the *meso*ITO electrode (film thickness ≈4.5 μm, Figure S2) in a solvent bath mixture containing **STEMPO**, and heating the solution to 70 °C under a N_2_ atmosphere for 6 h. Multi‐scan cyclic voltammetry (CV) measurements were used to deduce the optimal mixture, in which the surface loading of **STEMPO** (Γ_**STEMPO**_) on the *meso*ITO scaffold was both *maximal* and *stable*, with Γ_**STEMPO**_ being determined by integrating the charge passed in the oxidation wave of the consecutive cyclic voltammograms (see Supporting Information, Equation (S1)). The best mixture consisted of a **STEMPO** solution (2 mm) with 2 %(v/v) acetic acid (AcOH) and 1 %(v/v) H_2_O in acetonitrile (MeCN). With regards to the stability of the immobilized **STEMPO** compound, MeCN was the most suitable solvent from those attempted (Figure S3). The combination of AcOH and H_2_O facilitated the hydrolysis of the silatrane cage on the *meso*ITO surface,^28^ and was deemed necessary for instigating the anchoring process (Figure S4 and S5). Optimal Γ_**STEMPO**_ was typically found to be 20–25 nmol cm^−2^, which is in the expected range for nanostructured ITO surfaces.^29^, ^30^

**Figure 2:**
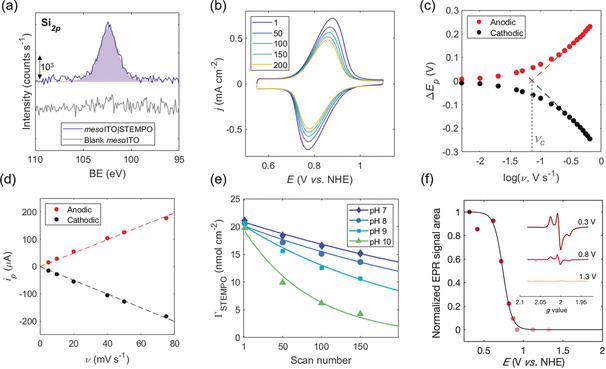
Characterization of *meso*ITO|**STEMPO**, assembled under optimized conditions. a) Si_*2p*_ XPS spectrum of *meso*ITO|**STEMPO**. b) Multi‐scan CV, conditions: pH 8 aq. HCO_3_^−^/CO_3_^2−^ (0.2 m), *ν*=50 mV s^−1^, N_2_, r.t. (legend denotes scan number). c) Trumpet plot deduced from the variable scan rate CV measurements; conditions: pH 8 aq. HCO_3_^−^/CO_3_^2−^ (0.5 m), N_2_, r.t. d) *i_p_* vs. *ν* plot, for *ν*<*ν_c_*. e) Stability curves as a function of pH (data fitted to a mono‐exponential decay), formulated by tracing the change in Γ_**STEMPO**_ (obtained through integration of the oxidation wave in the CV) over scan number in the multi‐scan CV experiment. f) FE‐EPR potentiometric titration of *C*‐*meso*ITO|**STEMPO**. Peak area of the **STEMPO** EPR signal as a function of potential (colored dots), fitted to 1 e^−^ Nernst equation (solid line). Inset: X‐band (9.5 GHz) EPR spectra of **STEMPO** at different applied potentials. Measurements performed at 100 K, 2 mW microwave power, 100 kHz modulation frequency and 2 G modulation amplitude.

X‐ray photoelectron spectroscopy (XPS) showed binding signals in the Si_*2p*_ and N_*1s*_ regions (Figure 2 a and Figure S6, respectively), where the Si_*2p*_ signal agrees with XPS reference spectra for the siloxane‐bearing group.^31^

Multiple CV scans of the *meso*ITO|**STEMPO** electrode reveal a reversible redox wave at *E*_1/2_=0.83 V vs. NHE (Figure 2 b), which corresponds to the nitroxide/oxoammonium species, and is only slightly more positive than that of dissolved TEMPO (*E*_1/2_=0.74 V vs. NHE, Figure S7a). At low scan rate (10 mV s^−1^), the peak‐to‐peak separation is below 20 mV and is thus in good agreement with the ideal value of 0 mV for a reversible response of a surface‐adsorbed species (Figure S7b). The full width at half‐maximum is 116 mV (Figure S7b), only slightly broader than the theoretically predicted value of around 91 mV for a 1 e^−^ process (at 25 °C).^32^ This slight deviation from ideal behavior can be attributed to multilayer formation,^33^, ^34^ stemming from the cross‐polymerization of Si‐O‐Si bonds between adjacent anchoring units in the mesoporous scaffold and film resistance of the *meso*ITO electrode.

A deeper analysis of the electron‐transfer dynamics of the *meso*ITO|**STEMPO** system was inferred using the Laviron method,^35^ which relies on the change in the peak potential (Δ*E*_p_) with scan rate (*ν*). The resulting trumpet plot for the *meso*ITO|**STEMPO** assembly is portrayed in Figure 2 c. The intercepts of the linear regions of the plot can be used to deduce the critical scan rate (*ν*_c_) and the apparent electron transfer rate constant (*k*_app_) for the system (see Supporting Information for further details). Values for *ν*_c_ and *k*_app_ were determined to be equal to 72±2 mV s^−1^ and 0.68±0.02 s^−1^, respectively. The rate of electron transfer appears to be low (hence the low value for *ν*_c_), but is comparable with other covalently linked redox species in the literature.^36^ Figure S8 depicts the CV scans measured over a range of scan rates to highlight the change in the peak‐to‐peak separation for the **STEMPO** redox wave as the applied scan rate exceeds *ν*_c_. The linear relationship between the peak current (*i*_p_) and *ν*, for *ν*<*ν*_c_ (Figure 2 d), is characteristic for a surface‐immobilized redox entity.^32^ The pH stability of the *meso*ITO|**STEMPO** assembly was investigated using a multi‐scan CV approach, whereby the electrode was subjected to several redox cycles in solutions of differing pH (Figure 2 e and Figure S9). A good stability was obtained after 200 scans at pH 7 and 8 (decrease in signal intensity of 34 % and 39 %, respectively, relative to scan 1), and the decay curve only began to be more severe at pH 10. These results support that the assembly is suited to operate under the basic conditions required for enhanced TEMPO catalysis.

Immobilization and direct wiring of **STEMPO** to the *meso*ITO electrode was confirmed by film electrochemical electron paramagnetic resonance (FE‐EPR) spectroscopy (see Supporting Information).^37^ The combined FE‐EPR spectroelectrochemical technique allows for the appearance and disappearance of paramagnetic species to be monitored as a function of the applied potential in the absence of any mediators. The high electrical conductivity combined with the surface‐modification properties of ITO make it a suitable platform for carrying out FE‐EPR spectroscopy. Carbon‐based electrodes tend to give rise to large radical signals and are thus unsuitable for such studies.^37^

For FE‐EPR spectroscopy, cylindrical *meso*ITO (*C*‐*meso*ITO) electrodes were employed for use in the EPR spectroelectrochemical cell. The unpaired electron in the TEMPO moiety is delocalized around the N and O atoms with nuclear spins (*I*) of 1 and 0, respectively, and thus only couples with N nuclei. This interaction gives rise to a triplet pattern in which the peaks, for the case of a diffusional species tumbling rapidly in solution at room temperature, are all the same intensity (EPR spectrum for diffusional TEMPO presented in Figure S10a, black trace). A triplet pattern is also discernible for the *C*‐*meso*ITO|**STEMPO** assembly, but the peak intensities are distorted in this case (Figure S10a, red trace). This change in line‐shape of the EPR spectrum relative to the diffusional case arises from a slower tumbling rate which can be a consequence of the impaired mobility of the TEMPO moiety upon **STEMPO** immobilization.^38^

Figure 2 f highlights the results from the FE‐EPR investigation. *C*‐*meso*ITO|**STEMPO** samples were poised at a particular potential, using a three‐electrode setup, and then flash‐frozen to allow for low‐temperature EPR characterization. Examples of EPR spectra, at three different potentials, are presented in Figure 2 f (inset) (full range in Figure S10b), where an increase in the applied bias is accompanied by a drop in signal intensity, that eventually vanishes due to the oxidation of the radical to EPR‐silent **STEMPO** ^+^. The shape of the EPR spectra for *E*<1.0 V vs. NHE are typical of nitroxide radicals measured at low temperatures (100 K).^39^ The normalized signal area of each individual EPR spectrum was plotted as a function of the potential, and is a close fit to the anticipated 1 e^−^ Nernst equation (solid line, Figure 2 f).

**Figure 3:**
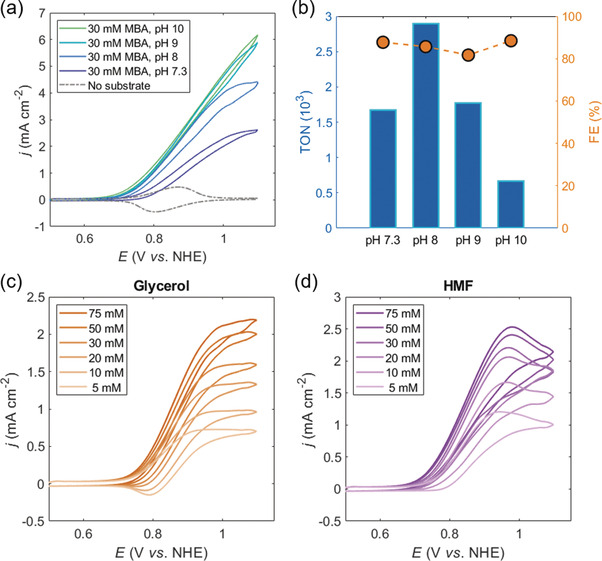
a) pH dependent CV scans for *meso*ITO|**STEMPO**, in the presence of 30 mm MBA. b) TON and FE metrics compiled from CPE experiments under a range of pH values. Conditions: pH 7.3: CO_2_ saturated aq. HCO_3_^−^/CO_3_^2−^ (0.5 m); pH 8–10: aq. HCO_3_^−^/CO_3_^2−^ (0.5 m) titrated under ambient conditions to the correct pH; for CV: *ν*=20 mV s^−1^, r.t.; for CPE: *E*_app_=1.0 V vs. NHE, *t*_CPE_=3 h, r.t., MBA (30 mm). Product quantification by HPLC was used for TON and FE calculation (Supporting Information, Equations (S4) and (S5)). Electrochemically determined concentration profiles for c) glycerol, and d) HMF; conditions: pH 8, *ν*=20 mV s^−1^, r.t.

Next, we investigated the catalytic performance of the *meso*ITO|**STEMPO** assembly. Figure 3 a depicts the catalytic behavior of the system as a function of the solution pH, where 4‐methylbenzyl alcohol (MBA) was chosen as a model substrate. The current density increases with increasing pH, accompanied by a lower onset potential for catalysis (from 0.75 V at pH 7.3, to 0.68 V at pH 10, vs. NHE), which is comparable to previous reports for immobilized TEMPO on carbon‐based electrodes.^40^, ^41^ This observation is in‐line with the established TEMPO‐mediated oxidation mechanism, whereby alcohol deprotonation leads to formation of a pre‐oxidation complex via nucleophilic attack of the alkoxide on the electrophilic nitrogen of the oxidized TEMPO moiety (the oxoammonium cation), prior to aldehyde formation.^24^, ^41^, ^42^, ^43^ However, the enhancement starts to plateau between pH 9 and pH 10, contrary to what is observed for TEMPO, and related nitroxyl derivatives, in solution.^43^ The plateau shown in Figure 3 a for the *meso*ITO|**STEMPO** system could be due to a combination of factors, and we rationalize this behavior to stem from the relatively slow electron transfer between the ITO electrode and immobilized **STEMPO**, as well as from mass transport limitations of the substrate in the mesoporous film.

Controlled potential electrolysis (CPE) was then conducted at an applied potential (*E*_app_) of 1.0 V vs. NHE at room temperature, to further probe the effect of pH on the *meso*ITO|**STEMPO** system. Figure 2 e shows that the *stability* of the anodic, molecular assembly is high at pH 7 and 8, but less so at pH 10. However, the TEMPO‐mediated catalysis, and hence reaction *kinetics*, are favored under more alkaline conditions (Figure 3 a). To compare the overall *meso*ITO|**STEMPO** performance as a function of pH, the TON and FE (Supporting Information, Equations (S4) and (S5), respectively) were calculated after a 3 h CPE experiment with MBA (30 mm) as the substrate, at four different pH values (Figure 3 b, Figure S11). The moles of product, 4‐methylbenzaldehyde (*n*_MBAd_), originating from selective MBA oxidation, were quantified by high performance liquid chromatography (HPLC) (Supporting Information).

The TON for **STEMPO** experiences a maximum at pH 8, reaching a value close to 3000, highlighting the fine balance between immobilization stability and catalytic activity in long‐term electrolysis experiments. On either side of the maximum, there is a corresponding decrease in the TON. At lower pH, this can be attributed to a lower rate of substrate oxidation thereby resulting in less *n*_MBAd_, whereas higher pH adversely affects the stability of the *meso*ITO|**STEMPO** assembly, likely leading to a loss of the catalytic sites from the electrode over reaction time. Post‐CPE (at pH 8) XPS conducted on the *meso*ITO|**STEMPO** electrode reveals peaks in the Si_*2p*_ and N_*1s*_ regions (Figure S12), similar to those observed on a freshly assembled electrode (Figure 2 a and Figure S6), and thus indicates that the gradual drop in activity could be primarily due to hydrolysis of the amide bond and subsequent loss of the TEMPO moiety from the assembly. On the other hand, the FE is invariant with the pH (average of 86±3 % as calculated across the pH range, Figure 3 b), implying that the charge passed at the electrode|catalyst interface is utilized in the same, selective manner (being directed towards substrate oxidation) throughout the pH range.

The versatility of the hybrid electrode was demonstrated by extending the substrate scope to glycerol, cellulose‐derived hydroxymethylfurfural (HMF), and the lignin model compound 2‐phenoxy‐1‐phenylethanol (PP‐ol; Table S2).^44^ A turnover frequency (TOF) analysis based on the sigmoidal catalytic response of the CV trace was performed for the **STEMPO** system in the presence of the different substrates (Supporting Information).^45^ Figures 3 c and 3d depict concentration profiles obtained for glycerol and HMF, respectively, and the concentration profile for MBA is shown in Figure S13a (corresponding “maximum current density vs. concentration” plots for these three substrates are presented in Figure S13b–d). PP‐ol was poorly soluble in pure aqueous electrolyte, and thus a CV trace for this compound was recorded in a MeCN–water mixture (Figure S14). The estimated TOFs for the four compounds, and the relevant experimental conditions, are summarized in Table S2. The results show that the *meso*ITO|**STEMPO** system can be used to oxidize a variety of alcohol‐based substrates, with the primary benzylic alcohols MBA and HMF showing the highest activity (TOF=0.677 and 0.680 s^−1^, respectively), followed by the aliphatic triol, glycerol (0.557 s^−1^). The results from this analysis therefore encourage the use of low‐cost and abundant alcohols such as glycerol for electrocatalytic applications with the *meso*ITO|**STEMPO** electrode. PP‐ol gave the lowest TOF (0.268 s^−1^), which agrees with the expected trend that primary alcohols are oxidized more rapidly than secondary alcohols by TEMPO in basic solution.^42^

Having characterized the anodic assembly and demonstrated the electrocatalytic compatibility of *meso*ITO|**STEMPO** with a variety of biomass representative alcohols, we turned towards applying this system within a coupled electrolyzer. Conversion of CO_2_‐to‐syngas as the cathodic half‐reaction presents an attractive strategy to utilize the electrons from alcohol oxidation by *meso*ITO|**STEMPO**. To facilitate a cost‐efficient redox cycle, use of robust, earth‐abundant catalysts for selective CO_2_R is essential. While many 3d transition metal‐based molecular catalysts have been developed over the years,^46^ **CoPc** has emerged as one of the most promising catalysts for CO_2_‐to‐CO reduction because of its enhanced performance upon immobilization onto polymers and carbon‐based electrodes.^47^, ^48^, ^49^ In the coupled electrolyzer, we employed a CNT‐**CoPPc** hybrid, fabricated by in situ polymerization, that was subsequently deposited on CP.^17^ The CP|CNT‐**CoPPc** cathode catalyzes the electrochemical reduction of CO_2_ to syngas, with a CO:H_2_ ratio dependent on the applied potential.^17^, ^50^

**Figure 4:**
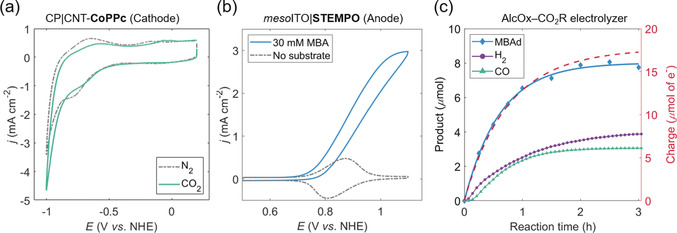
CV scans for the a) CP|CNT‐**CoPPc** cathode, and b) *meso*ITO|**STEMPO** anode, recorded separately in a three‐electrode setup, with Pt mesh as the CE and Ag/AgCl as RE. Conditions: CO_2_ saturated pH 7.3 aq. HCO_3_^−^/CO_3_^2−^ (0.5 m), *ν*=20 mV s^−1^, r.t. (pH 8 for cathode N_2_ trace). c) Coupled electrolyzer, showing product (liquid and gaseous) and charge passed (red trace, as recorded by the potentiostat, where 1 mol of e^−^=96,485 C or 1 faraday) over reaction time. Conditions: two‐compartment cell fitted with anion‐exchange membrane; three‐electrode configuration with *meso*ITO|**STEMPO** as WE, CP|CNT‐**CoPPc** as CE and Ag/AgCl as RE; CO_2_ saturated pH 7.3 aq. HCO_3_^−^/CO_3_^2−^ (0.5 m) in both compartments; MBA substrate (30 mm) present in anodic compartment, *E*_app_ (anode)=1.0 V vs. NHE, *t*_CPE_=3 h, r.t.; MBAd quantified by HPLC, CO/H_2_ by continuous flow GC.

The CV trace recorded for CP|CNT‐**CoPPc** under N_2_ displays a broad quasi‐reversible redox process (Figure 4 a, *E*_1/2_≈−0.71 V vs. NHE), which corresponds to the metal‐centered Co^II^/Co^I^ reduction of **CoPPc**. The surface concentration of electroactive cobalt centers was estimated to be 18.3±1.6 nmol cm^−2^ from integration of the Co^I^/Co^II^ oxidation wave (Figure S15). This corresponds to 5.6±0.5 % cobalt sites being electrochemically accessible, whereby the total amount of Co was determined using inductively coupled plasma optical emission spectroscopy (ICP‐OES) measurements (Supporting Information, Eq. (S6)).

A catalytic onset from the CP|CNT‐**CoPPc** electrode was observed in a CO_2_‐saturated solution at a potential close to −0.84 V vs. NHE (Figure 4 a). Electrocatalytic performance of the cathode was probed by stepped constant potential chronoamperometry in the range of −0.70 to −1.00 V vs. NHE, with 50 mV increments and 30 min steps (Figure S16). Product formation was monitored via a continuous flow gas chromatography (GC) method (Supporting Information). H_2_ was the only product until −0.80 V vs. NHE and CO evolution started at more negative potentials (≈−0.85 V vs. NHE). The selectivity of the electrode towards CO increases sharply at more negative potentials, reaching 76 % at −1.00 V vs. NHE (overpotential, *η*=0.46 V, where *E*(CO_2_/CO)=−0.54 V vs. NHE at pH 7.3).^51^ Within the same potential range, the blank CNT electrode did not generate any H_2_ or CO (Figure S16a, purple trace).

To elucidate the working principle of the coupled *meso*ITO|**STEMPO**–CP|CNT‐**CoPPc** electrolyzer, initial experiments were conducted using MBA. A catalytic wave for the *meso*ITO|**STEMPO** assembly in the presence of MBA (30 mm) was observed, which appeared to plateau at around 3 mA cm^−2^, at an applied potential just above 1 V vs. NHE (Figure 4 b). The *meso*ITO|**STEMPO** electrode displayed slightly lower current densities than CP|CNT‐**CoPPc** and was therefore selected as the working electrode (WE) in the coupled electrolyzer, while the cathode assumed the role of the counter electrode (CE). A two‐compartment electrochemical cell was employed with a Selemion‐AMV anion‐exchange membrane to separate the compartments. A Ag/AgCl reference electrode (RE) was placed in the working compartment and the three‐electrode configuration was adopted prior to studying a two‐electrode system, to be able to precisely control the *E*_app_ at the WE versus a known reference (Supporting Information). This also allowed us to record the exact potential at the CE (*E*_CE_) during electrolysis against the same reference, thus providing a more detailed description of the cell parameters over reaction time.

A CO_2_‐saturated carbonate buffer (0.5 m) was used in both compartments, which yielded a solution pH close to 7.3 that remained relatively constant throughout the experiment. Figure 4 c depicts the results from the coupled electrolysis (three‐electrode configuration), with *E*_app_=1.0 V vs. NHE at room temperature. Alcohol conversion to the corresponding aldehyde, MBAd, was quantified by HPLC, whereas CO and H_2_ were quantified by a continuous flow GC method (Supporting Information). Catalytic metrics obtained for the respective anode and cathode highlight the effectiveness of the combined system. MBA oxidation resulted in a TON_**STEMPO**_ of 1515 and FE close to 90 % after the 3 h CPE experiment. The TON_**STEMPO**_ was lower than expected from the TOF analysis from CV scans (Table S2) due to the modest stability of the anodic assembly, as demonstrated by the multiple CV scan measurements and prolonged CPE (cf. Figure 2 e and Figure 3 b, respectively). A cobalt‐based TON for syngas generation of 1360 (TON_CO_=599 and TON${}_{H{}_{2}}$ =761) and overall FEs for CO and H_2_ of 35 % and 45 %, respectively, were achieved for the CP|CNT‐**CoPPc** cathode.

**Figure 5:**
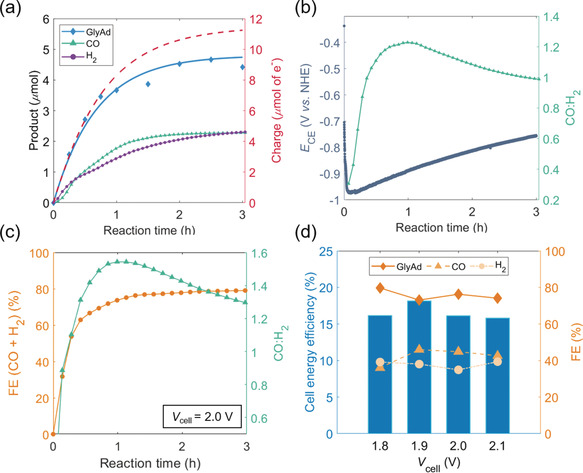
a) Similar profile to that shown in Figure 4 c, but using glycerol as the substrate (*E_app_* (anode)=1.0 V vs. NHE). b) Trend in the CO:H_2_ ratio and CE (i.e. CP|CNT‐**CoPPc**) potential (*E*_CE_) over reaction time. c) Combined FE for CO and H_2_, and CO:H_2_ ratio for the two‐electrode configuration employing glycerol as the substrate (applied cell potential=2.0 V). d) Cell energy efficiency and FE plotted as a function of *V*_cell_ in the same two‐electrode setup. Conditions: for both the three‐electrode (plots (a) and (b)), and two‐electrode (plots (c) and (d)) configurations, a two‐compartment cell (fitted with anion‐exchange membrane) was used; anode compartment: N_2_ saturated pH 8.3 aq. HCO_3_^−^/CO_3_^2−^ (0.5 m); cathode compartment: CO_2_ saturated pH 7.3 aq. HCO_3_^−^/CO_3_^2−^ (0.5 m); glycerol substrate (50 mm) present in anodic compartment, *t*_CPE_=3 h, r.t. Glycerol oxidation and gaseous products quantified by HPLC and continuous flow GC analysis, respectively.

This performance encouraged the substitution of MBA for glycerol, on account of its advantages as a potential substrate for coupling with CO_2_R in ‘real‐life’ applications. A similar setup to that used for coupled MBA oxidation was employed, except in this case, the anode compartment consisted of a carbonate buffer (0.5 m) at pH 8.3 (under N_2_), whereas the catholyte was comprised of a CO_2_ saturated carbonate buffer (0.5 m) at pH 7.3. This was deemed necessary for glycerol, as the **STEMPO**‐mediated catalysis involving this substrate was observed to be too sluggish at the quasi‐neutral pH of CO_2_‐saturated carbonate buffer (i.e. pH 7.3), but increased in activity under more alkaline conditions (as evidenced by the CVs recorded at pH 7.3 and 8.3, Figure S17). Figure 5 a illustrates the reaction time plot obtained with glycerol as the substrate, with *E*_app_=1.0 V vs. NHE. HPLC analysis revealed that glyceraldehyde (GlyAd) was the primary anodic product from the coupled electrolysis experiment.

The two compartments maintained their individual pH values for the duration of the electrolysis, and a TON_**STEMPO**_ and FE of 997 and 83 %, respectively, were measured for the anodic half‐reaction. Although precautions were taken to minimize overoxidation or further reaction of GlyAd, trace amounts of some side‐product can potentially form (not detected by HPLC), leading to the observed ≈7 % drop in the FE relative to the MBA electrolyzer. With regards to the cathode metrics, the cobalt‐based TON was determined to be equal to 894 (TON_CO_=444 and TON${}_{H{}_{2}}$ =450), while similar FEs for the gaseous products, relative to the MBA‐based electrolyzer, were measured (FE=41 % for CO, 41 % for H_2_). A side‐by‐side comparison of the calculated FEs for the liquid and gaseous products over reaction time, for the MBA‐ and glycerol‐based electrolyzers, is provided in the Supporting Information (Figure S18).

The three‐electrode configuration allowed for *E*_CE_ (i.e. the potential at the CP|CNT‐**CoPPc** electrode) to be monitored throughout the course of the electrolysis experiment. From the traces shown in Figure 5 b, there is an alteration in the CO:H_2_ ratio at the cathode over time, which seems to reflect the change in *E*_CE_. This decrease in the reducing potential at the cathode is itself a result of the gradual decline in activity at the anode over time. The change in the CO:H_2_ ratio as a function of the cathodic potential is in‐line with the stepped chronoamperometric experiments carried out for the CP|CNT‐**CoPPc** electrode (with Pt mesh as CE), as discussed above (Figure S16). The time‐lag between the minima of the *E*_CE_ trace and the maximum value of CO:H_2_ ratio on Figure 5 b is likely caused by the slow diffusion of CO from the porous cathode.

We furthered our investigation into coupled glycerol oxidation and CO_2_R, and performed a series of experiments in a more practical two‐electrode configuration, while varying the applied cell potential (*V*_cell_). Values for *V*_cell_ in the range of 1.8 to 2.1 V were chosen, based on the rationale that: |*E*_cathode_−*E*_anode_|≈|${\overset{-}{E}}_{\mathrm{CE}}$ −*E*_app_|=1.85 V, where ${\overset{-}{E}}_{\mathrm{CE}}$ is the average potential at the CE, over reaction time, as measured in the three‐electrode configuration (i.e. Figure 5 b). Figure 5 c depicts the combined FE at the cathode (for CO and H_2_) and the CO:H_2_ ratio, over reaction time, for *V*_cell_=2.0 V. The trends agree with those obtained for the three‐electrode setup. The increase in the maximum of the CO:H_2_ ratio for the two‐ versus three‐electrode configuration (shown in Figure 5 b) could be a result of the increased driving force provided by the 2.0 V potential. This bias most likely leads to more reductive potentials at the cathode, and, in accordance with the stepped chronoamperometry data for CP|CNT‐**CoPPc** (Figure S16), would translate to a higher CO:H_2_ ratio.

Finally, we calculated the cell energy efficiency (*ϵ*), as a function of *V*_cell_ using Equation (1):^12^

(1) $$\epsilon =\frac{\left|E_{\mathrm{cell}}\right|}{V_{\mathrm{cell}}}=\frac{\left|\left({\mathrm{FE}}_{H_{2}}E_{H^{+}{/H}_{2}}+{\mathrm{FE}}_{\mathrm{CO}}E_{{\mathrm{CO}}_{2}/\mathrm{CO}}\right)-{\mathrm{FE}}_{\mathrm{GlyAd}}E_{\mathrm{GlyAd}/\mathrm{glycerol}}\right|}{V_{\mathrm{cell}}}$$

where $E_{H^{+}{/H}_{2}}$ , $E_{{\mathrm{CO}}_{2}/\mathrm{CO}}$ , and *E*_GlyAd/glycerol_ denote the reduction potentials for H^+^, CO_2_, and glyceraldehyde, respectively, under non‐standard conditions (Table S3). A more detailed breakdown regarding the thermodynamic analysis required to compute *ϵ* is provided in the Supporting Information. Figure 5 d illustrates the FEs for the anodic and cathodic processes, along with the corresponding *ϵ* calculations, for different *V*_cell_ values. There is a slight improvement in the CO selectivity upon increasing from 1.8 to 1.9 V (FE_CO_=36 and 46 %, respectively), presumably a result of the higher driving force at these applied voltages. This enhancement is met with an improvement in *ϵ* (from 16 to 18 %), since the 100 mV additional bias is offset by the increase in FE_CO_, as governed by Equation (1). However, for *V*_cell_≥2.0 V, the combined effects of a largely unchanged CO:H_2_ ratio and anodic FE, causes a corresponding drop in the cell efficiency to ≈16 %, similar to that obtained for *V*_cell_=1.8 V. The cell energy efficiency values measured for our hybrid electrolyzer are in accordance with those reported in the literature, where for example an efficiency of 17 %, at 1.8 V cell potential, was measured for a dual electrolyzer featuring benzyl alcohol oxidation coupled with the reduction of aqueous CO_2_ to CO and H_2_.^9^ However, the previously reported system was comprised of Ru‐based molecular catalysts for the reductive and oxidative half‐reactions, and additionally, only one of the catalysts was immobilized. In contrast, we have incorporated immobilized cathodic and anodic catalysts in our electrolyzer, both free of any precious metals, and have also demonstrated the applicability of the tandem AlcOx–CO_2_R device to couple the oxidation of more commercially viable substrates, such as glycerol, with CO_2_‐to‐syngas conversion.

## Conclusion

We have designed, fabricated and characterized an anode featuring a silatrane‐modified TEMPO molecule on a *meso*ITO scaffold, and demonstrated the electrocatalytic ability of the molecularly engineered MO_*x*_ system to efficiently oxidize a variety of biomass representative substrates. The siloxane anchor, formed upon hydrolysis of the silatrane cage on the MO_*x*_ surface, displays robust binding. The catalytically active site (i.e. the oxoammonium cation) is both stable and readily regenerated under electrocatalytic conditions,^52^ and we believe that the long‐term stability of the hybrid electrode assembly is currently limited by the amide bond in **STEMPO**. Improvements to the molecular design of the linker employed for **STEMPO** will provide a possibility to enhance the stability and overall activity of the anodic assembly.

We further showed the advantage and versatility of our novel **STEMPO** anode by coupling alcohol oxidation with an efficient CO_2_R cathode (CP|CNT‐**CoPPc**), to construct an AlcOx–CO_2_R electrolyzer based on immobilized, precious‐metal‐free, ‘molecular’ catalysts. The functionality and performance of the device was investigated using a three‐electrode configuration, first employing MBA as a model substrate, and later, using the commercially applicable substrate, glycerol. It was found that in both cases, stoichiometric amounts of a selective oxidation product (the corresponding aldehyde) and syngas were generated at the anode and cathode, respectively. FEs were typically excellent for the hybrid system, exceeding 80 % for both anode and cathode. TONs were also high, approaching 1000 for *meso*ITO|**STEMPO** and 900 for CP|CNT‐**CoPPc** (with glycerol as substrate). The TON of the cathode in the electrolyzer is currently limited by the prolonged stability issue of the anodic assembly during continuous CPE experiments and the **CoPPc**‐cathode on its own is known to maintain activity over a longer time‐period.^17^ Further studies were then made using a demonstrator‐type, two‐electrode setup for coupled glycerol oxidation at the anode and syngas generation at the cathode, showing similar performance metrics as the three‐electrode system. Cell energy efficiency calculations also revealed the advantages of operating at a lower *V*_cell_, with a maximum efficiency of 18 % being measured at a cell potential of 1.9 V. This molecular hybrid system is therefore a suitable model for the development of future AlcOx–CO_2_R electrolyzers based on earth‐abundant materials, which can provide chemical feedstocks (aldehydes and syngas) from sustainable and abundant resources, such as biomass‐derived alcohols, CO_2_, and renewable electricity.

## Conflict of interest

The authors declare no conflict of interest.

## Supporting information

As a service to our authors and readers, this journal provides supporting information supplied by the authors. Such materials are peer reviewed and may be re‐organized for online delivery, but are not copy‐edited or typeset. Technical support issues arising from supporting information (other than missing files) should be addressed to the authors.

Supplementary
