# Supplementary material for: A Precious‐Metal‐Free Hybrid Electrolyzer for Alcohol Oxidation Coupled to CO2‐to‐Syngas Conversion
Source: Angew Chem Int Ed Engl. 2020 May 18;59(36):15633–41. doi: 10.1002/anie.202002680 (PMC7496929; doi:10.1002/anie.202002680)
Supplement: Supplementary file 1 — Supplementary [file ANIE-59-15633-s001.pdf]

Angewandte Chemie
Eine Zeitschrift der Gesellschaft Deutscher Chemiker

Supporting Information

A Precious-Metal-Free Hybrid Electrolyzer for Alcohol Oxidation Coupled to CO_2-to-Syngas Conversion

*Mark A. Bajada[+], Souvik Roy[+], Julien Warnan[+], Kaltum Abdiaziz, Andreas Wagner, Maxie M. Roessler, and Erwin Reisner**

anie_202002680_sm_miscellaneous_information.pdf

Supporting Information

Experimental Section

Materials

All chemicals purchased from commercial suppliers were of the highest available purity and used without further purification unless otherwise noted. ITO-coated glass substrates were purchased from Vision Tek Systems Ltd. (R = 12 Ω sq^{-1}, thickness = 1.1 mm). Ti foil (0.25 mm thick, 99.5%), 4-methylbenzyl alcohol (MBA), 4-metylbenzaldehyde (MBAd) 1,8-Diazabicyclo(5.4.0)undec-7-ene (DBU), and Carbon black (Super P® Conductive, product # H30254) were purchased from Alfa Aesar. Anhydrous ethanol (200 proof, ≥ 99.5%), triethanolamine, 5-hydroxymethylfurfural, multi-walled carbon nanotubes, and ITO nanoparticles (< 50 nm) were purchased from Sigma Aldrich. Sodium carbonate (anhydrous), potassium bicarbonate (anhydrous), 3-aminopropyl(triethoxy)silane, and thionyl chloride were purchased from Acros Organics, 1,2,4,5-tetracyanobenzene, 2-phenoxy-1-phenylethanol, glyceraldehyde (GlyAd), and DL-glyceric acid (20% in water) from Fluorochem, and 4-carboxy-TEMPO from Insight Biotechnology. Untreated carbon paper (AvCarb® MGL190) was purchased from Fuel Cell Earth. Glycerol was purchased from VWR Chemicals, and anhydrous pyridine, diisopropylethylamine, and glacial acetic acid from Fisher Scientific.

All aqueous solutions were prepared with ultrapure water (DI water; Milli-Q®, 18.2 MΩ cm). Toluene, dichloromethane (DCM), hexane, pentane, and acetonitrile (MeCN) were distilled before use.

Physical Characterization

^{1}H NMR spectra were recorded on a Bruker 400 MHz or 500 MHz DCH cryoprobe spectrometer at room temperature. Chemical shifts for ^{1}H NMR spectra were referenced to residual signals from the deuterated solvent. High resolution-mass spectra (HRMS) were recorded using a Thermo Scientific Orbitrap Classic mass spectrometer. FT-IR (ATR) spectra were recorded on a Thermo Scientific Nicolet iS50 spectrometer. Raman spectra were recorded on a HORIBA LabRAM HR Evolution

system with an incident laser of 633 nm. Elemental analysis was carried out by the Microanalysis Service of the Department of Chemistry, University of Cambridge, using an Exeter Analytical CE-440 Elemental Analyzer. Inductively coupled plasma optical emission spectrometry (ICP-OES) measurements were also conducted by the Microanalysis Service, on a Thermo Scientific iCAP 7400 ICPOES DUO spectrometer. The surface morphology of electrodes was analyzed using a Tescan MIRA3 FEG-SEM. X-ray photoelectron spectroscopy (XPS) was carried out on an ESCALAB 250Xi Thermo Fisher Scientific spectrometer, operating in the constant analyzer energy mode. An Al-Kα X-ray source was used with power of 124 W (8.5 mA and 14.55 kV), and the pass energy was set for the recorded data at 20 eV to acquire high-resolution spectra. All spectra were calibrated, and charge compensation was applied. The spectra were referenced to 284.8 eV C_{1s} peak (adventitious carbon impurities).

Synthesis

Synthesis of 3-aminopropylsilatrane (1). 3-aminopropylsilatrane was synthesized according to a literature procedure.[1–3] 3-aminopropyl(triethoxy)silane (5.00 g, 22.5 mmol, 1.05 equiv.) was added to triethanolamine (3.18 g, 21.375 mmol, 1 equiv.) at room temperature in a round bottomed flask fitted with a Dean Stark apparatus under N_2. The resulting mixture was refluxed at 145 °C to remove the ethanol formed during the reaction. Once the ethanol was completely removed, the reaction mixture was allowed to cool to room temperature, and was then placed in the fridge for 1 h. A white solid was obtained which was then washed with hexane, filtered, dried under vacuum, and stored in the fridge. Characterizations were found to be similar to previously published data and the product was used in the next step without further purification.

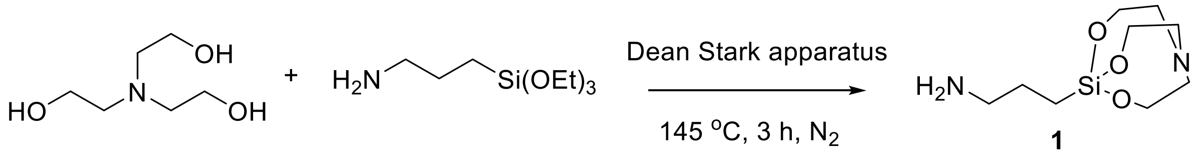

Synthesis and characterization of STEMPO. Pyridine (0.24 mL, 3 mmol, 2.4 equiv.) was added to a solution of 4-carboxy-TEMPO (250 mg, 1.25 mmol, 1 equiv.) in anhydrous toluene (12.5 mL) under N_2. The resulting solution was cooled to 0 °C in an ice bath and thionyl chloride (0.14 mL, 1.875 mmol, 1.5 equiv.) was added dropwise. The solution was stirred at room temperature for 1 h and an off-white

precipitate was observed. The liquid layer was transferred into a round bottom flask, and the precipitate was washed with a small amount of anhydrous toluene. The combined liquid fractions were concentrated *in vacuo* and the resulting residue dissolved in anhydrous dichloromethane (DCM) (5 mL). This crude product was added to a stirring solution of **1** (348.5 mg, 1.5 mmol, 1.2 equiv.) and *i*-Pr_2NEt (0.4 mL, 2.3 mmol, 1.8 equiv.), dissolved in anhydrous DCM (10 mL) under N_2 atmosphere. The resulting mixture was then stirred at room temperature for 1 h under N_2. The reaction mixture was concentrated, and the product was precipitated from DCM by the addition of n-pentane. The solvent was syphoned off, and the orange solid was dried under vacuum. The resultant orange powder was solubilized in DCM (30 mL) and washed successively with 1% $NaHCO_3$ water (20 mL) and brine (20 mL). The organic layer was dried over $MgSO_4$ and filtered over cotton. The solvent was evaporated under reduced pressure, and the orange powder was dried overnight under vacuum to afford **STEMPO** (70% yield). FT-IR (ATR): σ (cm^{-1}): 2972, 2929, 2878, 1646, 1547, 1456, 1178, 1123, 1086. HRMS (+ESI, m/z): calcd. for $C_{19}H_{37}O_5N_3Si_1$ $[M-H^+]^+$: 415.2502; found, 415.2496. Anal. calcd. for $C_{19}H_{36}O_5N_3Si_1$: C, 55.04; H, 8.75; N, 10.14; found: C, 55.21; H, 8.80; N, 9.69.

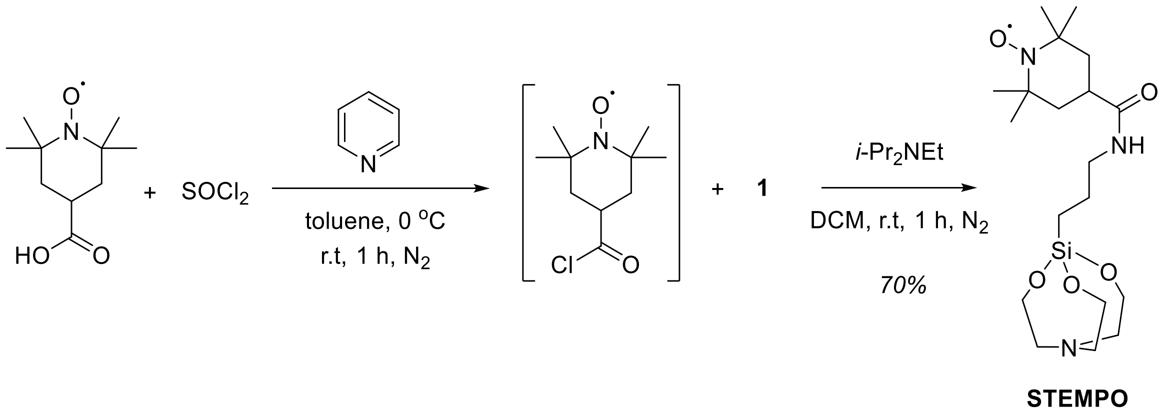

Electrode Assembly

Fabrication of *meso*ITO electrodes. *meso*ITO electrodes were fabricated in-house following a modified literature procedure.[4,5] For *meso*ITO, 3×1 cm ITO-coated glass slides were sonicated in acetone and isopropanol for 15 min each, and then dried in an N_2 stream. A 5 wt.% mixture of ITO nanopowder in an acetic acid solution (5 M in ethanol) was sonicated for 20 min. Parafilm was used to define a constant surface

area on the ITO-coated glass (0.25 cm^2), onto which the ITO dispersion was drop-cast. Two 5 µL aliquots were dropped onto the defined area, with an intermediate drying period (in air) of 5 min. Following the second drying period, the parafilm template was removed and the samples annealed in a Carbolite furnace, under atmospheric conditions, using the following heating program: the slides were heated from room temperature to 450 ºC (ramp rate of 4 ºC min^{-1}), kept at this temperature for 20 min, and then slowly cooled to room temperature in the furnace chamber.

Assembly of *meso*ITO|STEMPO electrodes. Three organic solvents were chosen for the initial '**STEMPO** loading' optimization on the *meso*ITO electrode – ethanol (polar and protic), MeCN (polar and aprotic), and toluene (non-polar and aprotic). Solutions of each (2 mM **STEMPO**, 2.8 mL), containing 0.2% v/v AcOH, were prepared, and the ITO|*meso*ITO electrodes were submerged in the solution in a capped vial, and heated at 70 °C under a N_2 overpressure for a duration of 6 h. Multi CV measurements revealed that MeCN offered better stability in comparison to ethanol and toluene (Figure S3), and hence was the solvent of choice for further optimization. The effect of AcOH and H_2O concentration was then probed by varying the AcOH/H_2O ratio, until a maximum in **STEMPO** surface loading was observed – 2% v/v AcOH and 1% v/v H_2O in MeCN provided the best conditions for the system at hand (Figure S4 and S5). All further *meso*ITO|**STEMPO** electrodes were assembled using these conditions, unless otherwise stated.

Fabrication of CP|CNT-CoPPc electrodes. The polymerization of cobalt phthalocyanine (**CoPc**) onto carbon nanotube fibers (CNT) to form the CNT-polymeric **CoPc** composite (CNT-**CoPPc**; where **CoPPc** denotes *polymeric cobalt phthalocyanine*) was carried out in accordance to a literature procedure.[6] For fabrication of the cathodes, a catalyst ink was prepared by dispersing 2 mg of the hybrid CNT-**CoPPc** material and 1 mg conductive carbon black in 0.5 mL ethanol containing 0.012 mL 10 wt% Nafion solution, followed by ultrasonication for 30 min. 0.05 mL of catalyst ink containing ~0.2 mg CNT-**CoPPc** was drop-casted onto untreated carbon paper (CP, 1.6×0.8 cm^2) and dried under air, to yield the CP|CNT-**CoPPc** electrode assembly. The geometric area of the catalyst coverage was ~0.3 cm^2. An electric wire was connected to the carbon paper using a conductive silver paint (RS® Components 186-3593). The electrode was encapsulated with an opaque

grey epoxy adhesive (Loctite® EA 3423), leaving only the catalyst-covered area exposed (~0.28 cm^2).

Quantification of Cobalt. Cobalt loading in the hybrid CNT-**CoPPc** composite was determined by ICP-OES after digestion of the material (< 1 mg) in conc. HNO_3 (70%) (~1 mL) overnight and dilution to 10 mL with Milli-Q® water. To quantify the cobalt content of CP|CNT-**CoPPc** electrodes by ICP-OES, the catalyst powder (mixture of CNT, **CoPPc**, and carbon black) was scratched off the electrode after electrocatalysis and digested in conc. HNO_3 followed by dilution to 10 mL.

Electrochemical Studies.

(a) General Methodology. Cyclic voltammetry (CV) and controlled potential electrolysis (CPE) were performed on either an Ivium CompactStat potentiostat or a BioLogic VSP potentiostat. When investigating the oxidative or reductive half-reaction, a three-electrode configuration was employed in an airtight, one-compartment cell (solution volume = 9 mL), with a *meso*ITO|**STEMPO** working electrode (WE) or CP|CNT-**CoPPc** WE respectively, a Pt mesh counter electrode (CE) and a Ag/AgCl reference electrode (RE; for aqueous experiments: BASi RE-6, saturated KCl; for MeCN-water experiments: home-made RE in a solution with the same composition as the electrolyte). For aqueous experiments, the potentials were converted from Ag/AgCl to normal hydrogen electrode (NHE) by adding +0.199 V. In MeCN-water mixtures, the reference electrode was regularly referenced against the ferrocene couple (Fc^+/Fc), and potentials were reported against Fc^+/Fc. All electrochemical experiments were carried out at room temperature (~22 °C) unless otherwise stated.

The surface coverage/concentration of **STEMPO**, $\Gamma_{\mathbf{STEMPO}}$, was calculated through the integration of the oxidation wave in the CV scan:

$$\Gamma_{\mathbf{STEMPO}} = \frac{Q}{zFA_{anode}} \tag{S1}$$

where *Q* is the charge obtained from integration of the oxidation wave, *z* the number of electrons involved in the oxidation of the nitroxyl radical to the oxoammonium cation (i.e. $z = 1$), *F* is the Faraday constant, and A_{anode} is the geometrical area of the anode

(0.25 cm^2). The surface concentration of electroactive cobalt centers, Γ_{Co}, was determined using a similar technique (see Figure S15 for more details).

Unless otherwise stated, for fully aqueous conditions, a carbonate buffer (0.5 M) was employed. For pH 8, 9, and 10 solutions, the pH was adjusted by addition of H_2SO_4 under ambient conditions. Solutions were purged with N_2 for 15 min prior to measurement, to remove atmospheric O_2. Purging the carbonate buffer (0.5 M) with CO_2 for 30 min resulted in a solution pH of 7.3. A 3:2 H_2O:MeCN (0.3 M bicarbonate) was used for MeCN-water mixtures.

(b) Coupled electrolyzer. For the coupled electrolyzer set-up, a custom-made, airtight, two-compartment electrochemical cell was employed for all CPE measurements, where a Selemion-AMV anion-exchange membrane was utilized to separate the compartments. A three-electrode configuration was used with *meso*ITO|**STEMPO** as WE, CP|CNT-**CoPPc** as CE and Ag/AgCl as RE, with the WE and RE being placed in the same compartment. The solution volume of the working (anode) compartment was 7.5 mL, and the counter (cathode) compartment was 5 mL. When a two-electrode configuration was employed (by connecting the reference cable to the CE), the volume of the anode compartment was 8 mL.

Both compartments were stirred during the CPE measurement, and the duration of the CPE experiment was set to 3 h. When MBA was used as the organic substrate, both compartments contained carbonate buffer (0.5 M) and were purged with CO_2 for 30 min (pH 7.3 under CO_2 saturation). The cathode compartment was continuously purged with CO_2 (5 sccm) throughout the duration of the experiment, as required for continuous flow GC analysis (see below). When glycerol was employed as organic substrate, the anode compartment was purged with N_2 for 15 min to maintain a pH value close to 8 during the coupled CPE experiment. The cathode compartment was kept under similar conditions to those described above.

(c) Gaseous Product Analysis & Quantification. The quantification of H_2 and CO was performed with a Shimadzu Tracera GC-2010 Plus gas chromatograph equipped with a barrier discharge ionization detector. A Hayesep D (2 m × 1/8'' o.d. × 2 mm i.d., 80/100 mesh, Analytical Columns) precolumn and a RT-Molsieve 5A (30 m × 0.53 mm i.d., Restek) main column were used to separate H_2, O_2, N_2, CH_4 and CO while blocking CO_2 and H_2O from the sensitive Molsieve column. The He (5.0, BOC) carrier

gas was purified (HP2-220, VICI) prior to entering the GC. The column temperature was kept constant at 85 °C, the detector temperature was 300 °C.

The electrolyte was continuously purged with CO_2 (5 sccm) and the gas from the electrolysis cell was constantly flushed through a loop (1 mL) and injected every *ca.* 4.25 min into the GC. The GC was calibrated with a known standard for H_2, CO and CH_4 (2040 ppm H_2 / 2050 ppm CO / 2050 ppm CH_4 in balance gas CO_2, BOC, ± 2% grade) by diluting the gas with pure CO_2. The total Faradaic yield was generally observed to be below unity, which was presumably due to (i) slow release of bubbles from the porous electrode surface, and (ii) charging of the CNTs. The rates of gas evolution ($\dot{n}_{CO}$ or $\dot{n}_{H_2}$ mol s^{-1}) were calculated by means of the equation (S2):

$$\dot{n}_{CO/H_2} = \frac{Area_{GC}}{f} \cdot \frac{p}{RT} \cdot \dot{V} \tag{S2}$$

where f is the response factor determined by GC calibration, p is the pressure in the cell (ambient pressure), R is the universal gas constant, T is the temperature (298 K), and $\dot{V}$ the volumetric flow rate. The total amount of gas evolved was calculated by integrating the rate of CO and H_2 formation (mol s^{-1}) over time (s).

(d) Liquid Product Analysis & Quantification. During and after the reaction, a 50 µL aliquot of the solution was taken from the electrochemical cell and diluted with 450 µL of Milli-Q water (for experiments involving MBA) or 50 µL of a 0.55 M H_2SO_4 solution (for experiments involving glycerol), and then analyzed via high-performance liquid chromatography (HPLC). Substrate conversions and yields were deduced using a Shimadzu LC-20, with an ultraviolet-visible detector (Shimadzu SPD-10AV) set at 190 nm.

Conditions for MBA oxidation: samples and standards (1 µL) were injected directly onto a 150 mm × 4.6 mm Prodigy™ 3 µm ODS-3 100 Å column purchased from Phenomenex. The mobile phase was comprised of a 1:1 MeCN/H_2O mixture with a total flow rate of 0.5 mL min^{-1} at 40 °C. Typically, a 50 µL aliquot was sampled from the reaction mixture and diluted by a factor of 10 prior to HPLC analysis.

Conditions for glycerol oxidation: samples and standards (15 µL) were injected into a configuration comprised of a Security-Guard Carbo-H cartridge and a 300 mm × 7.8 mm Rezex™ ROA-Organic Acid H+ (8%) column, both purchased from Phenomenex. A dilute sulfuric acid (2.5 mM) solution was used as the eluent, and the

flow rate was set to 0.5 mL min^{-1} at 75 °C. For glycerol analysis, 50 µL aliquot was sampled from the anode compartment and quenched with 0.55 M H_2SO_4 (50 µL), to neutralize the carbonate solution and prevent the release of CO_2 inside the HPLC column.

When conducting either analysis, one hour of equilibration was required before the first sample injection. Initially, the starting materials and expected main products were analyzed separately to identify their respective retention times on the chromatogram. This was carried out for MBA, MBAd, glycerol, and GlyAd. Standard calibration curves of the main product compounds (MBAd and GlyAd, Figure S19) were then produced to afford product quantification and deduce the concentration of species in the reaction aliquot.

Chromatograms for standard solutions of common oxidation products from glycerol oxidation (namely: dihydroxyacetone, glyceric acid, and glycolic acid) were also compiled, but these compounds were not observed during analysis of the CPE reaction aliquots. Exemplary chromatograms from the coupled CPE experiment are presented in Figure S20.

EPR and FE-EPR spectroscopy

(a) Electrochemical measurement. 'Cylindrical *meso*ITO' (*C*-*meso*ITO) electrodes were prepared following a previously published protocol.[7] Electrochemical experiments were carried out using the standard three-electrode configuration consisting of the *C*-*meso*ITO electrode as the WE, teflon-insulated Ag wire as pseudo-RE, and bare Pt wire as CE. All electrochemical experiments were performed in an anaerobic glovebox (MBraun, < 0.05 ppm O_2) in buffer (20 mM Na_2CO_3, adjusted to pH 8) at room temperature. In the potentiometric titration, nine *C*-*meso*ITO**|STEMPO** electrodes were poised at different potentials until a stable current was reached, which occurred in less than 5 min, before the sample was flash frozen in a dry ice/acetone bath. The *C*-*meso*ITO structure was found to be unstable to repeated freeze-thaw cycles. A new electrode was therefore assembled for each spectroelectrochemical measurement, given that such measurements had to be conducted on frozen samples. All redox potentials are quoted against NHE, where $E_{\mathrm{NHE}} = E_{\mathrm{Ag\ wire}} + 0.220$ V. The

reference electrode (pseudo Ag wire) was calibrated using the redox potential of ferricyanide (pH 7) against a saturated calomel electrode (SCE).

(b) EPR spectroscopic measurements and analysis. Low-temperature EPR measurements were performed in the Centre for Advanced ESR (CAESR) located in the Department of Chemistry of the University of Oxford, using an EMXmicro X-band CW spectrometer (Bruker BioSpin GmbH, Germany), equipped with a helium flow cryostat (ESR900, Oxford instruments) and a rectangular resonator module with 10 mm sample access (X-band Super High Sensitivity Probehead, ER 4122SHQE). Room-temperature EPR measurements were carried out in the MRSH at Imperial College, using a Bruker EMX WinEPR CW spectrometer operating at X-band frequencies and equipped with rectangular resonator module with 10 mm sample access (X-band Super High Sensitivity Probehead, ER 4122SHQE). EPR measurements were conducted under non-saturating conditions with 2 mW microwave power, 9 scans, 100 kHz modulation frequency and 2 G modulation amplitude.

The spectra of the empty resonator and of samples containing only buffer (including only the glass tube) were found to be identical. For CW measurements, the Q value, as reported by the built-in Q indicator in the Xepr program (typically Q = 1500 ± 100), was used as a guide to position each sample in the same location in the resonator. All data analysis was carried out using EasySpin.[8] To obtain the potentiometric titration curve, the area of each EPR spectrum was obtained through double integration, and plotted against its corresponding potential. The experimental data points were then fitted to the 1 e^- Nernst equation.

Supporting Calculations and Tables

1. Laviron Analysis

The two equations employed to carry out the Laviron analysis for the *meso*ITO|**STEMPO** assembly are as follows:[9]

$$\Delta E_{p,a} = \frac{-2.3RT}{(1-\alpha)zF}\log\left(\frac{(1-\alpha)zF}{RTk_{app}}\right) - \frac{2.3RT}{(1-\alpha)zF}\log(\nu) \qquad \text{(S3.a)}$$

$$\Delta E_{p,c} = \frac{-2.3RT}{\alpha zF}\log\left(\frac{\alpha zF}{RTk_{app}}\right) - \frac{2.3RT}{\alpha zF}\log(\nu) \qquad \text{(S3.b)}$$

where $\Delta E_{p,a}$ and $\Delta E_{p,c}$ denote the difference between the potential of the anodic peak (subscript *a*) and the formal redox potential ($E°'$), and that of the cathodic peak (subscript *c*) and $E°'$, respectively. $E°'$, for *meso*ITO|**STEMPO,** was obtained by averaging the anodic and cathodic potentials at low scan rates, ν. z is the number of electrons transferred, α is the electron-transfer coefficient, and k_{app} the apparent rate constant for electron-transfer. R, T, and F are the ideal gas constant, absolute temperature, and Faraday constant, respectively.

To apply the Laviron analysis, a linear trend was fitted to the anodic and cathodic regions of the trumpet plot portrayed in Figure 2c, for values of $\Delta E_{p,a}$, $\Delta E_{p,c}$ > 100 mV. $(1 - \alpha)$ and α were determined from the gradients of the anodic and cathodic trends, respectively, while $k_{app,a}$ and $k_{app,c}$ were deduced from the *y*-intercept of the corresponding plots. A value for k_{app} was afforded by taking the average of the two rate constants. The critical scan rate, ν_c, was obtained from the *x*-intercept, by extrapolating the linear portions of the trumpet plot in both regions to $\Delta E_p = 0$. As per k_{app}, ν_c was then calculated based on the average between the anodic and cathodic values.

2. Catalyst Metrics

The turnover number (TON) was calculated using:

$$\textit{Anode}: \text{TON}_{\textbf{STEMPO}} = \frac{n_{\text{Aldehyde}}(\text{HPLC})}{n_{\textbf{STEMPO}}\ (\text{EC})} \quad \text{(S4.a)}$$

$$\textit{Cathode}: \text{TON}_{\text{Co}} = \frac{n_{\text{CO+H}_2}(\text{GC})}{n_{\text{Co}}\ (\text{EC})} \quad \text{(S4.b)}$$

while the Faradaic efficiency (FE) was obtained using:

$$\textit{Anode}: \text{FE (Aldehyde)} = \frac{n_{\text{Aldehyde}}(\text{HPLC})}{Q_{CPE}/2F} \quad \text{(S5.a)}$$

$$\textit{Cathode}: \text{FE (CO + H}_2) = \frac{n_{\text{CO+H}_2}(\text{GC})}{Q_{CPE}/2F} \quad \text{(S5.b)}$$

where the moles of catalyst, $n_{\textbf{STEMPO}}$ for the anode and n_{Co} for the cathode, were determined electrochemically (EC) (see equation (S1) and Figure S15), while the moles of liquid product (n_{Aldehyde}) was determined via HPLC, and moles of gaseous product ($n_{\text{CO+H}_2}$) via continuous flow GC. Q_{CPE} signifies the charge passed during the CPE experiment, and the factor of ‘2’ in the FE equation denotes a 2 e^- process.

3. ICP Data

Table S1: Quantification of Co loaded onto the surface of CP|CNT-**CoPPc** electrodes, as measured by ICP-OES. Loadings are given per geometric surface area.

Sample [a]	Weight (mg)	[Co] (ppm)	Total Co (mg)	Total Co (µmol) [b]	Average Co loading (nmol cm^{-2})
CNT-**CoPPc**	0.63	6.29	0.0629	-	-
CP|CNT-**CoPPc** (i)	-	0.54	0.0054	0.091	327±5
CP|CNT-**CoPPc** (ii)	-	0.54	0.0054	0.092	
CP|CNT-**CoPPc** (iii)	-	0.55	0.0055	0.093	
CP|CNT-**CoPPc** (iv)	-	0.53	0.0053	0.089	

[a] CP|CNT-**CoPPc** (i)-(iv) denote four separate electrodes from a single batch.
[b] Total Co loading on the CP|CNT-**CoPPc** electrode ($A_{cathode}$ = 0.28 cm^2).

For cathode assembly:

$$\% \text{ electroactive Co} = \frac{\Gamma_{\text{Co}}(\text{EC})}{\text{Co loading (ICP)}} = \frac{18.3 \pm 1.6}{327 \pm 5} = 5.6 \pm 0.5\% \qquad \text{(S6)}$$

4. TOF Analysis

The turnover frequency (TOF) analysis for the heterogenous catalytic system, *meso*ITO|**STEMPO**, in the presence of different alcohol substrates, was carried out using the rationale outlined by Savéant.[10] The model of interest as described by the authors is applicable to a well-defined molecular catalyst with a well-defined standard potential, where the catalyst is deposited on the electrode surface; immobilized **STEMPO** is thus a suitable fit. In the paper, they assumed a fast electron transfer between the electrode and catalytically active redox couple, and based on the Nernst law and substrate behavior in the diffusion-convection layer, present a complete derivation to describe the catalyst TOF. The relevant equations required for our analysis are shown below:

$$\text{TOF}_{\textbf{STEMPO}} = \frac{k_r c^{o}_{sub}}{1 + \exp\left[{}^{F}/_{RT}\left(E - E^{o}_{\text{ST}^{+}/\text{ST}^{\bullet}}\right)\right]} \qquad \text{(S7.a)}$$

$$\frac{i_{max}}{A_{anode}F} = \frac{2k_r c^{o}_{sub}\Gamma_{\textbf{STEMPO}}}{1 + \exp\left[{}^{F}/_{RT}\left(E - E^{o}_{\text{ST}^{+}/\text{ST}^{\bullet}}\right)\right]} \Rightarrow \text{TOF}_{\textbf{STEMPO}} = \frac{i_{max}}{2A_{anode}F\,\Gamma_{\textbf{STEMPO}}} \qquad \text{(S7.b)}$$

where c^{o}_{sub} is the initial substrate concentration, k_r the apparent rate constant for the catalytic reaction, $i_{\max}$ the catalytic maximum current, $\Gamma_{\textbf{STEMPO}}$ the surface concentration of **STEMPO**, and $E^{o}_{\text{ST}^{+}/\text{ST}^{\bullet}}$ the standard potential of the **STEMPO** ('ST') redox couple. Hence, as shown in equation (S7.b), a value for the TOF can be calculated based on empirical data. It is worth noting that, as discussed, for the case of **STEMPO**, the apparent electron transfer kinetics are sluggish. To circumvent this issue, experimentally derived CVs were recorded using a slightly low scan rate (20 mV s^{-1}), to ensure maximal oxidation of the **STEMPO** surface species. This yields a Nernstian response, which can be fitted with a sigmoidal function in accordance with equation (S7) (cf. Figure 3c and 3d, Figure S13a, and Figure S14). Variation of the i_{max} was also less pronounced for $c^{o}_{sub} > 30$ mM, and hence, 30 mM was chosen as the substrate concentration for the TOF analysis (cf. maximum current density *vs.*

concentration plots in Figure S13). 20 mM PP-ol was selected due to the solubility issues of this substrate at higher concentrations in the mixed solvent. The results obtained from the TOF analysis for the four alcohol substrates are summarized in Table S2 below.

Table S2. TOF analysis of representative alcohol substrates.[a]

Substrate	Predicted TOF (s^{-1})
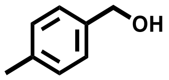	0.677
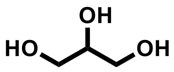	0.557
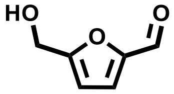	0.680
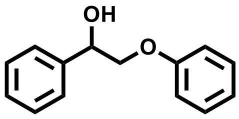	0.268

[a] Experimental conditions for TOF analysis: CV recorded under N_2, r.t, $\nu = 20$ mV s^{-1}, substrate concentration = 30 mM, pH 8 aq. HCO_3^- /CO_3^{2-} buffer (0.5 M). For the case of PP-ol, concentration = 20 mM, 3:2 H_2O:MeCN mixture, 0.3 M $KHCO_3$ electrolyte.

5. Cell Energy Efficiency

The following thermodynamic analysis was carried out in order to calculate ε, the cell energy efficiency, as defined by equation (1). The cathode and anode half-reactions can be expressed by the following:

$$\textit{Anode}: C_3H_8O_3 + 2OH^- \rightarrow C_3H_6O_3 + 2H_2O + 2e^- \quad \text{(S8.a)}$$

$$\textit{Cathode}: CO_2 + H_2O + 2e^- \rightarrow CO + 2OH^-$$
$$2H_2O + 2e^- \rightarrow H_2 + 2OH^- \quad \text{(S8.b)}$$

Using the standard potentials (E^o) available in the literature, the reduction potentials under the operating conditions of the coupled electrolyzer (E) were calculated (see Table S3). Where necessary, the standard Gibbs free energy change for the reaction of intertest, ΔG^o_{rxn}, was related to E^o through:

$$\Delta G^{o}_{rxn} = -\,nFE^{o} \quad \text{(S9)}$$

The Nernst law was also utilized for the thermodynamic calculation, where the shift in E^{o} with pH is given by:

$$E = E^{o} - 0.059 \cdot \mathrm{pH} \quad \text{(S10)}$$

Table S3. Reduction potential calculations under operating conditions of the two-electrode configuration.

Reduction potential, E (V *vs.* NHE)	Calculation	Reference values [a]
E(GlyAd/glycerol)	$\Delta G^{o}_{rxn} = \Delta G^{o}_{f}(\text{glycerol}) - G^{o}_{f}(\text{GlyAd})$ $= -77.38$ kJ mol^{-1}<br>$E = 0.4$ V – (0.059 × 8.3) = –0.0897	ΔG^{o}_{f}(glycerol) = –478.6 kJ mol^{-1} [11]<br>ΔG^{o}_{f}(GlyAd) = –401.2 kJ mol^{-1} [12]
$E(CO_2/CO)$	E = –0.106 V – (0.059 × 7.3) = –0.537	E^{o} (CO_2/CO) = –0.106 V *vs.* RHE [13]
$E(H^+/H_2)$	E = 0 V – (0.059 × 7.3) = –0.431	

[a] Values for glycerol and GlyAd reported under standard conditions of 1 bar and 298 K.

Supporting Figures

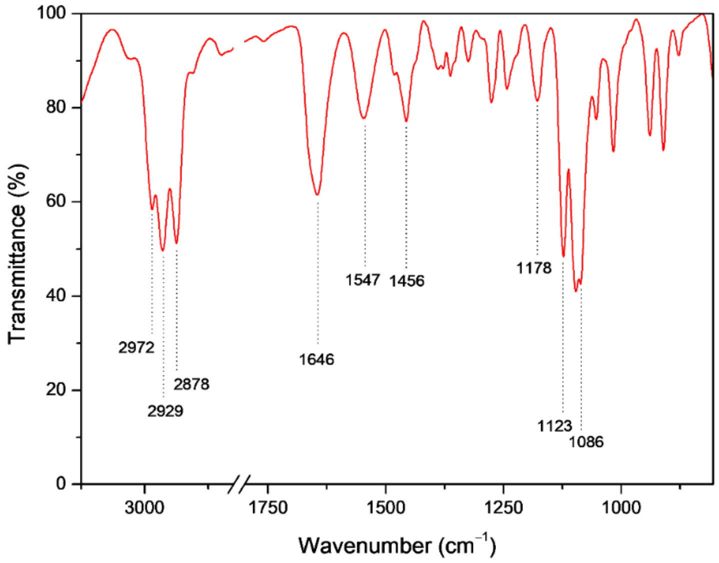

Figure S1. ATR-FTIR spectrum for **STEMPO**.

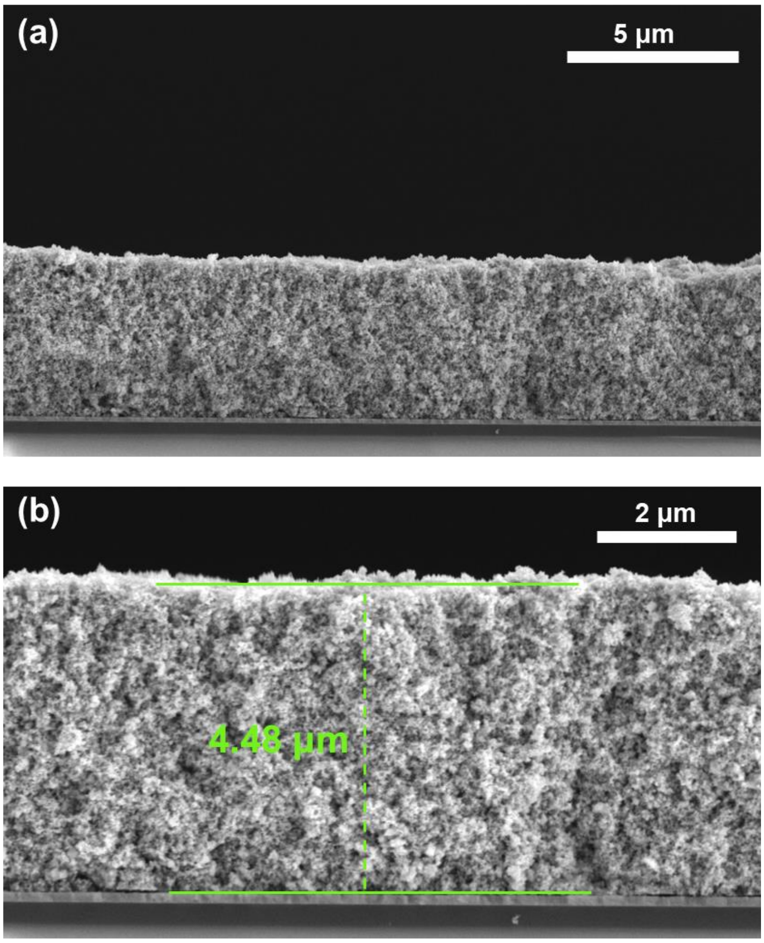

Figure S2. SEM cross-section image of the *meso*ITO electrode, at (a) 10.0 kx magnification, and (b) 20.0 kx magnification.

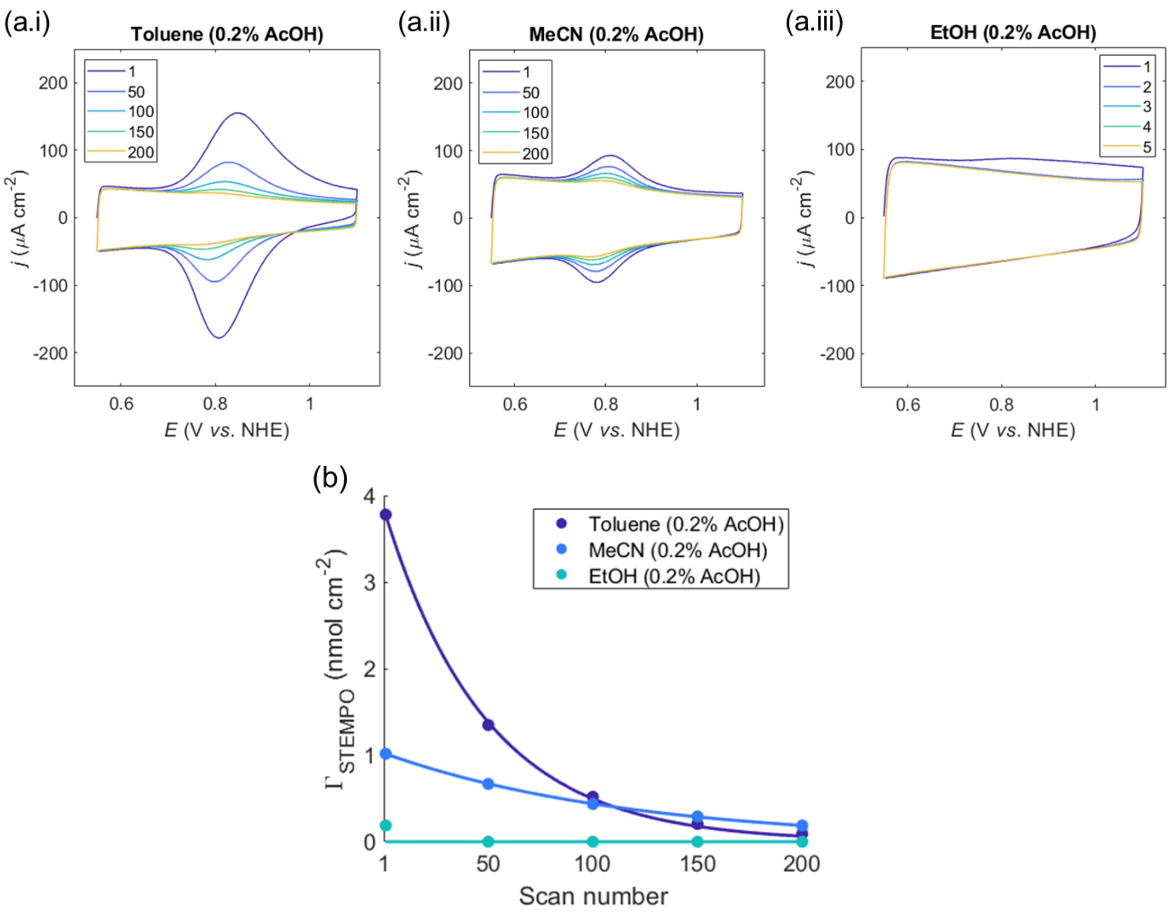

Figure S3. Effect of solvent on *meso*ITO|**STEMPO** assembly. (a) Multi-scan CVs, *conditions*: pH 8 aq. HCO_3^-/CO_3^{2-} (0.2 M), $\nu = 50$ mV s^{-1}, N_2, r.t (the legend denotes scan number). (b) Corresponding **STEMPO** surface loading versus scan number.

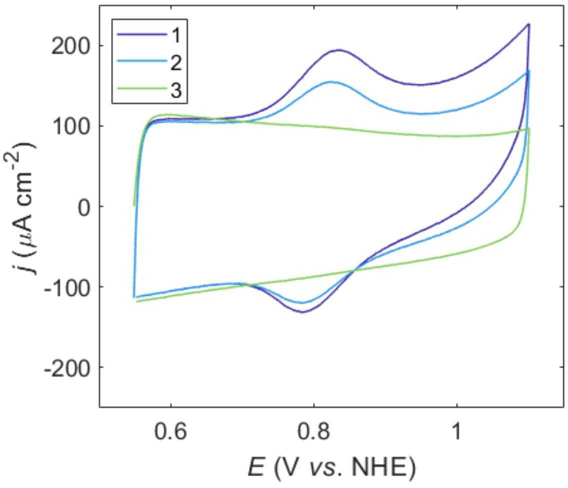

Figure S4. CV for *meso*ITO|**STEMPO**, assembled without any AcOH or H_2O additives (i.e. anhydrous MeCN solvent, 70 °C, N_2 overpressure, 6 h). *Conditions*: pH 8 aq. HCO_3^-/CO_3^{2-} (0.2 M), $\nu = 50$ mV s^{-1}, N_2, r.t (the legend denotes scan number).

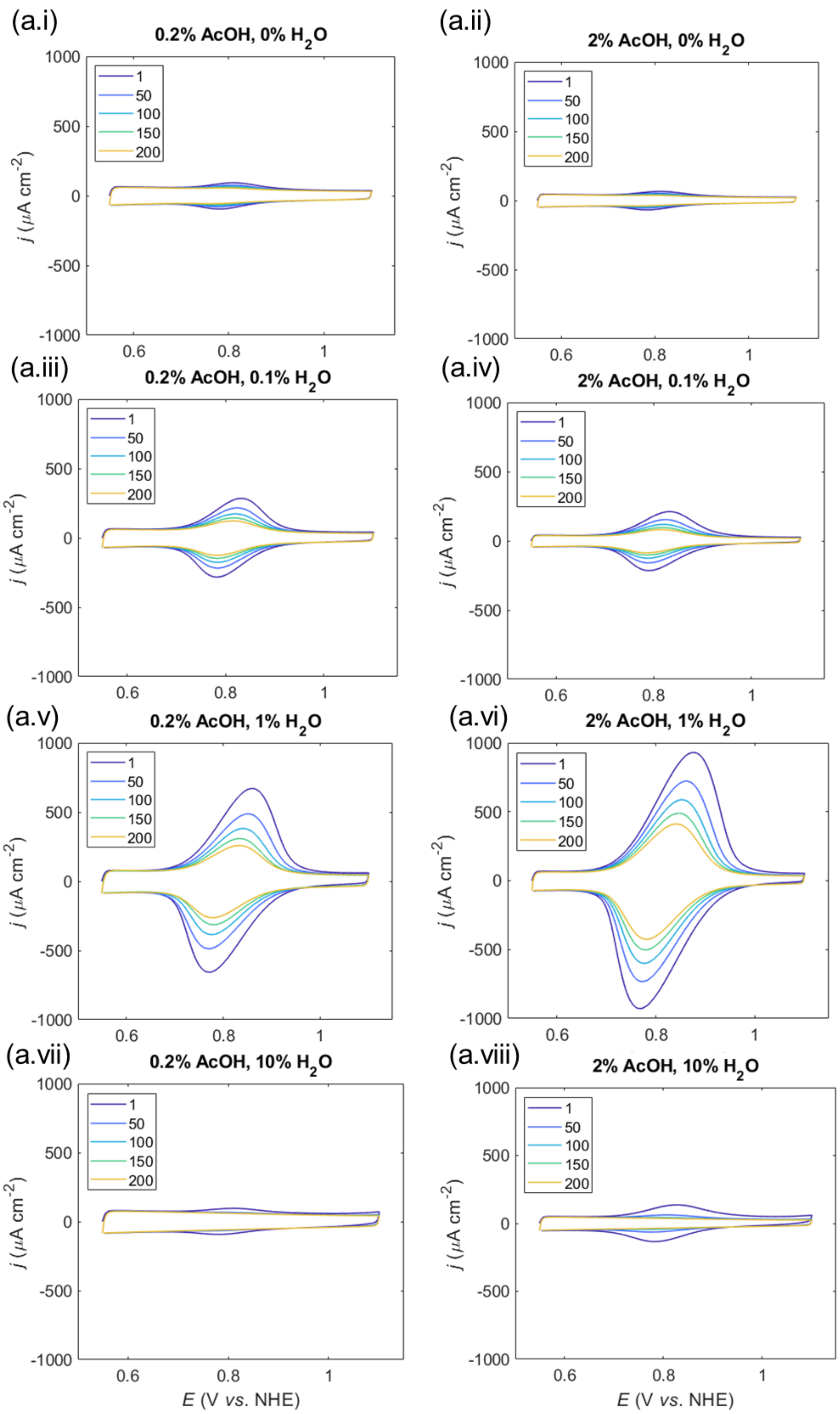

(a.i)
0.2% AcOH, 0% H2O
(a.ii)
2% AcOH, 0% H2O
(a.iii)
0.2% AcOH, 0.1% H2O
(a.iv)
2% AcOH, 0.1% H2O
(a.v)
0.2% AcOH, 1% H2O
(a.vi)
2% AcOH, 1% H2O
(a.vii)
0.2% AcOH, 10% H2O
(a.viii)
2% AcOH, 10% H2O
1
50
100
150
200
j (μA cm-2)
E (V vs. NHE)

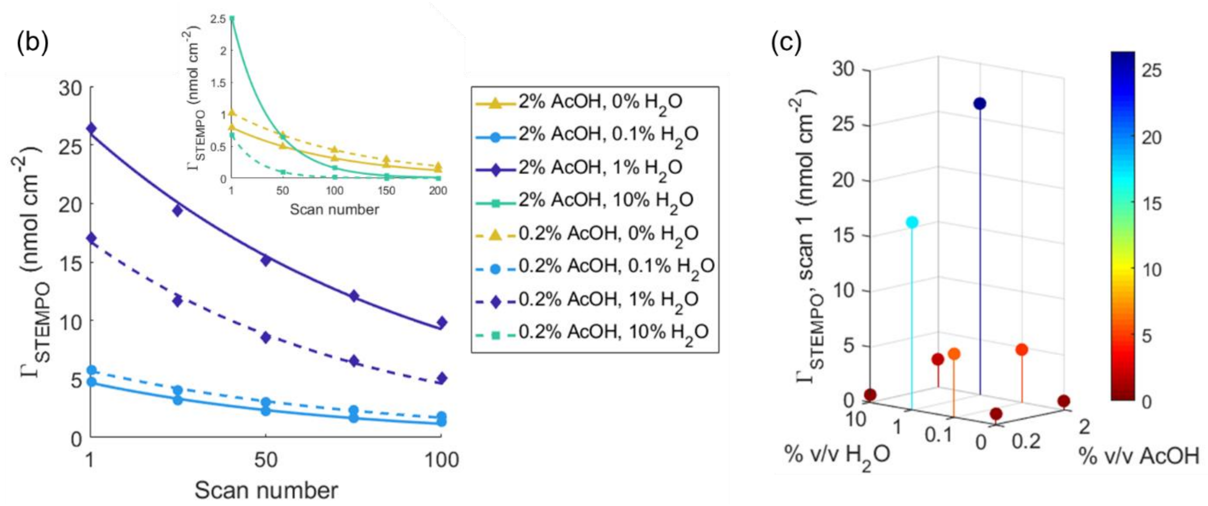

Figure S5. Effect of AcOH and H_2O concentration on *meso*ITO|**STEMPO** assembly. (a) Multi-scan CVs, *conditions*: pH 8 aq. HCO_3^-/CO_3^{2-} (0.2 M), $\nu = 50$ mV s^{-1}, N_2, r.t (the legend denotes scan number). (b) Corresponding **STEMPO** surface loading versus scan number, (c) **STEMPO** loading for scan 1, as a function of %AcOH and $\%H_2O$.

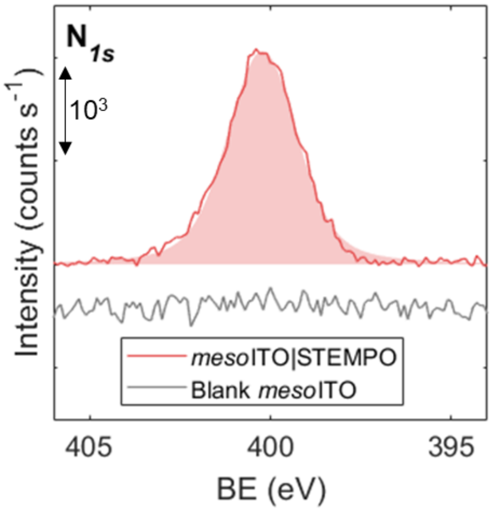

Figure S6. N_{1s} XPS spectrum for *meso*ITO|**STEMPO** (signal for blank *meso*ITO also shown).

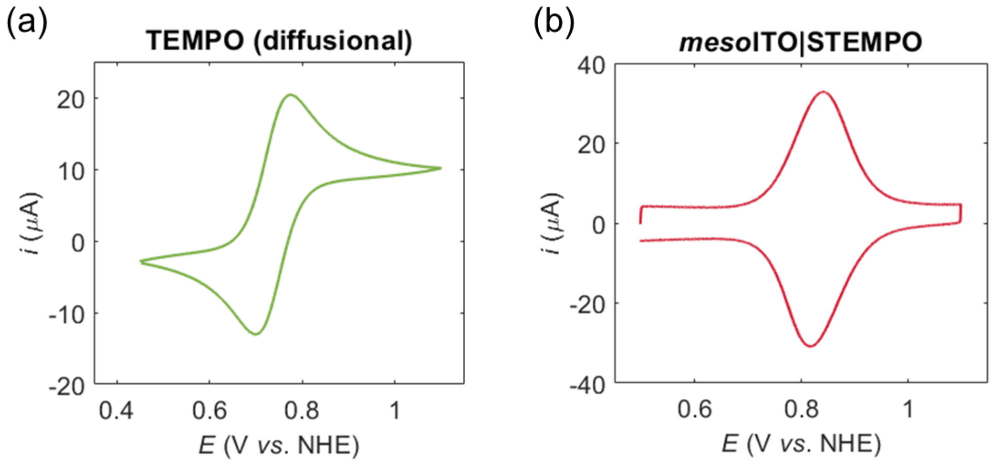

Figure S7. CVs recorded for (a) diffusional TEMPO (5 mM, glassy carbon working electrode), and (b) *meso*ITO|**STEMPO** (assembled under optimized conditions). *Conditions*: pH 8 aq. HCO_3^- /CO_3^{2-} (0.5 M), ν = 10 mV s^{-1}, N_2, r.t.

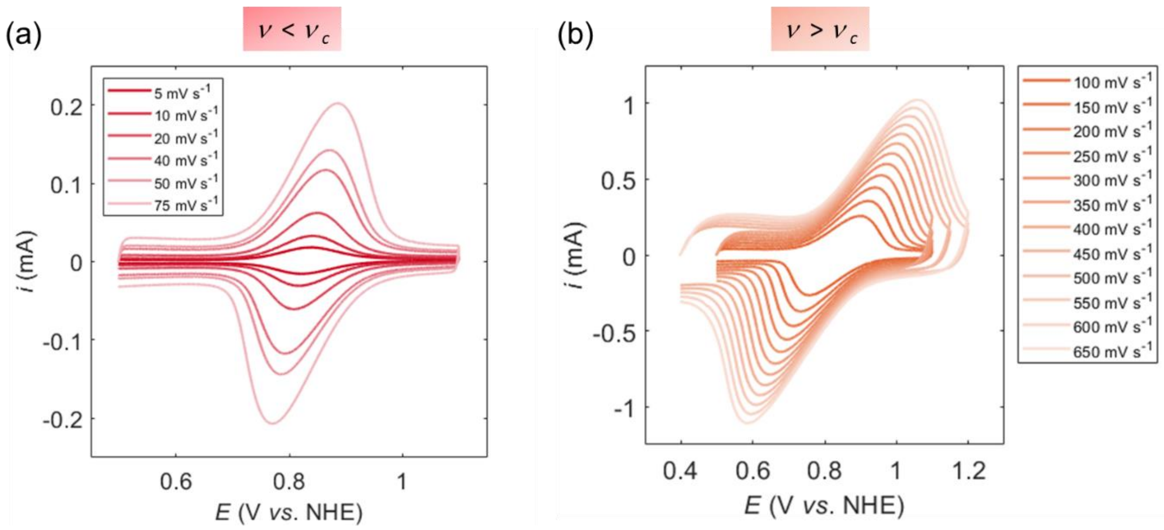

Figure S8. Variable scan rate CV, (a) below, and (b) above the critical scan rate. The variable scan rate assessment is required to be able to carry out the Laviron analysis and determine electron-transfer parameters for the *meso*ITO|**STEMPO** system.

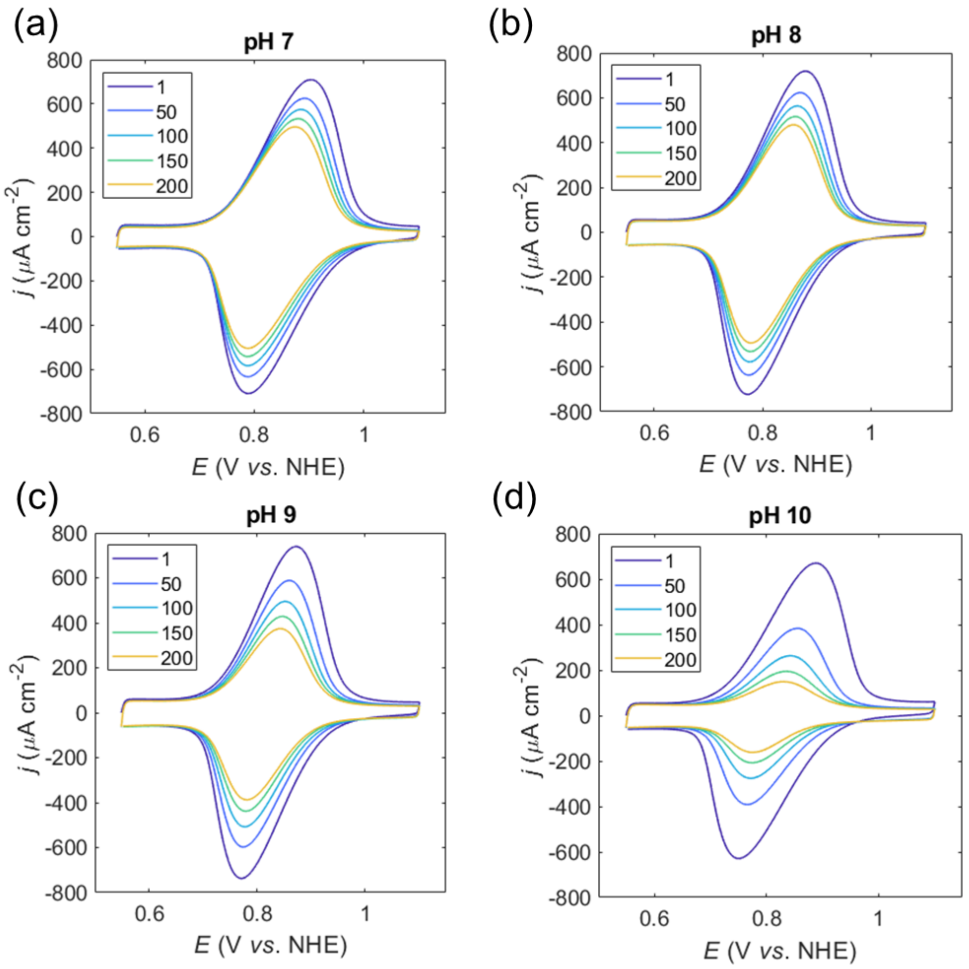

Figure S9. Multi-scan CVs for the *meso*ITO|**STEMPO** assembly recorded under different pH values; (a) pH 7 aq. Na_2SO_4 (0.1 M), (b), pH 8 aq. HCO_3^- /CO_3^{2-} (0.2 M), (c) pH 9 aq. HCO_3^- /CO_3^{2-} (0.2 M), (d) pH 10 aq. HCO_3^- /CO_3^{2-} (0.2 M). *General conditions*: ν = 50 mV s^{-1}, N_2, r.t (the legend denotes scan number).

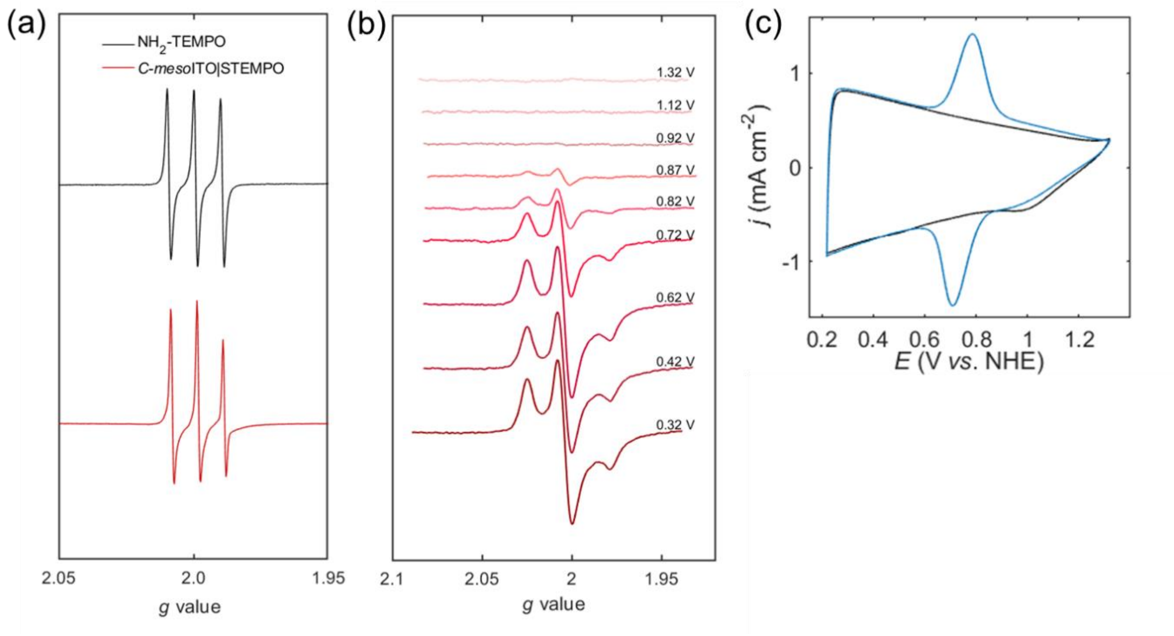

Figure S10. (a) X-band EPR spectra of diffusional NH_2-TEMPO and *C-meso*ITO|**STEMPO**; *conditions*: temperature = 298 K, 20.02 mW microwave power, 9 scans, and 1 G modulation amplitude. (b) Complete set of EPR spectra recorded for the *C-meso*ITO|**STEMPO** assembly, poised at different potentials (as specified on the plot); *conditions*: temperature = 100 K, 2 mW microwave power, 9 scans, and 2 G modulation amplitude. (c) CV for blank *C-meso*ITO (black trace) and *C-meso*ITO|**STEMPO** (blue trace); *conditions*: ν = 10 mV s^{-1}, air, temperature = 298 K. Note: pH 8 aq. HCO_3^- /CO_3^{2-} (0.2 M) was used in all cases, (a) – (c).

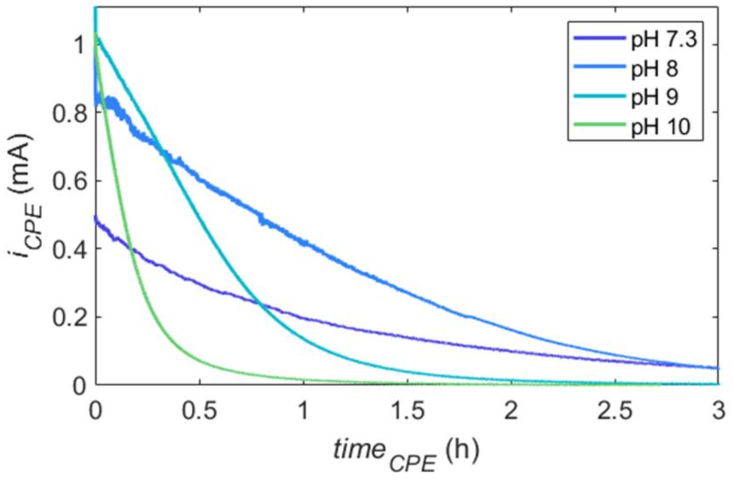

Figure S11. Chronoamperometric profiles recorded for the *assembly stability vs. kinetics* study as a function of pH, using *meso*ITO|**STEMPO** as the WE in a standard three-electrode configuration, with Pt as CE and Ag/AgCl as RE.

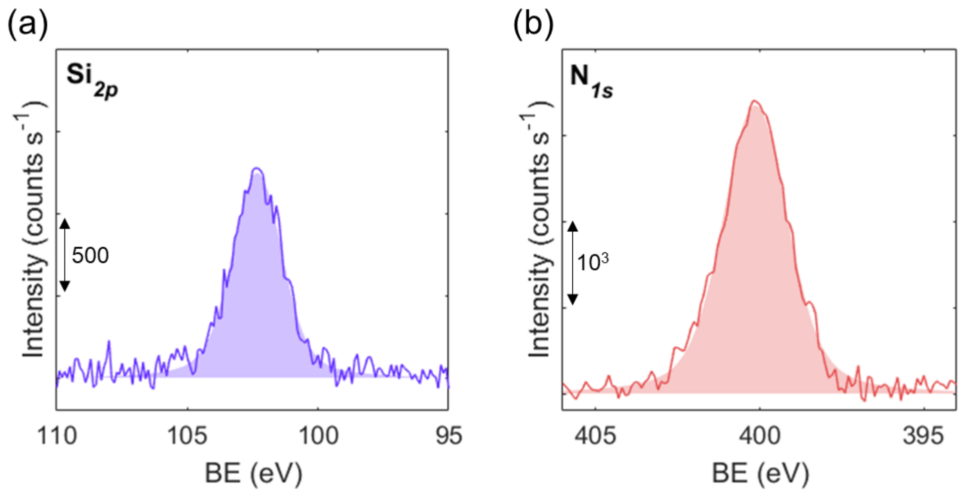

Figure S12. Post-CPE XPS measurements on *meso*ITO|**STEMPO**, for (a) Si_{2p} region and (b) N_{1s} region. (CPE conditions: E_{app} = 1 V *vs.* NHE, t_{CPE} = 3 h, pH 8 aq. HCO_3^- /CO_3^{2-} (0.5 M), N_2, r.t).

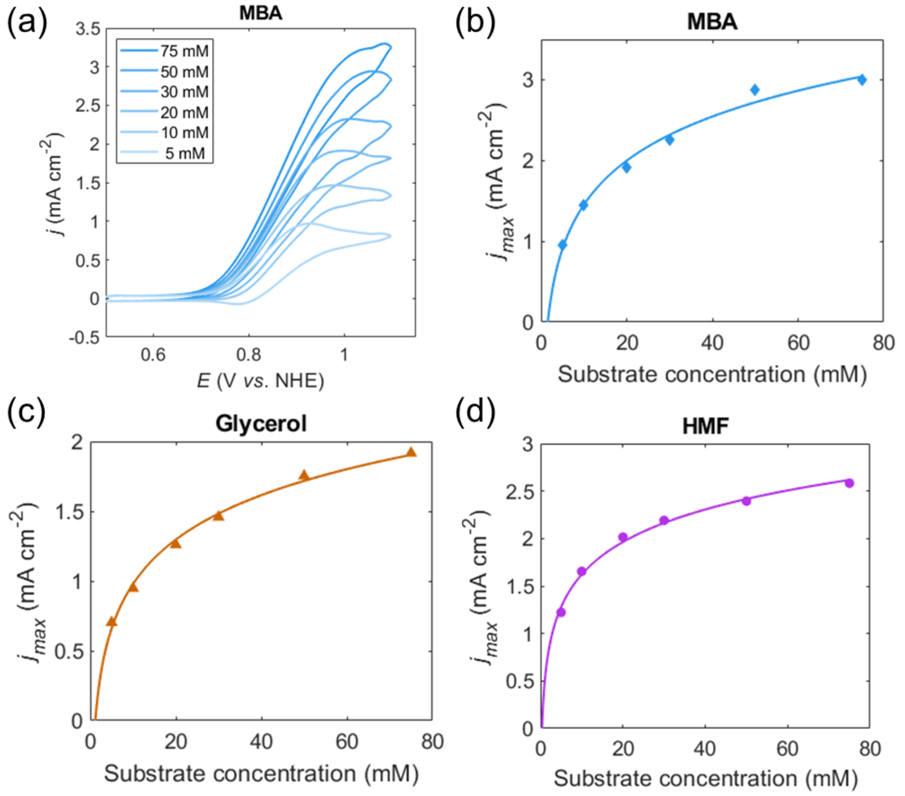

Figure S13. (a) Concentration profile for MBA substrate; *conditions*: pH 8 aq. HCO_3^- /CO_3^{2-} (0.5 M), ν = 20 mV s^{-1}, N_2, r.t. Maximum current density *vs.* concentration plots compiled for (b) MBA, (c) glycerol, and (d) HMF, based on the corresponding substrate concentration profiles. The trend line was fit using the equation: $j_{max} = \lambda_1 + \lambda_2 \ln(c^{o}_{sub})$, where λ_1 and λ_2 are substrate dependent coefficients for the fit, and c^{o}_{sub} denotes the initial substrate concentration.

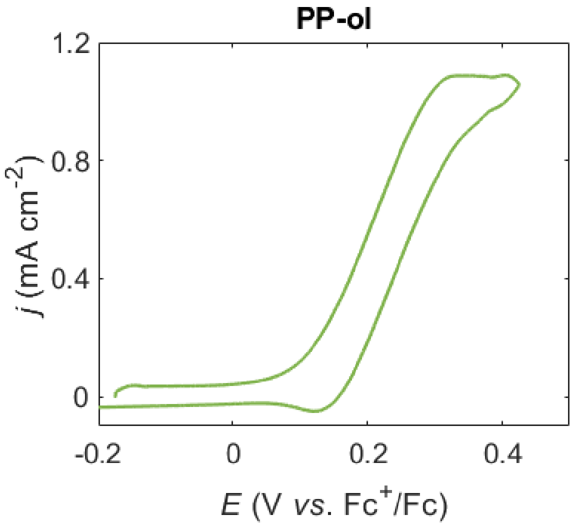

Figure S14. CV trace for PP-ol (20 mM), used for TOF analysis. *Conditions*: 3:2 H_2O:MeCN (0.3 M $KHCO_3$) solution, ν = 20 mV s^{-1}, N_2, r.t.

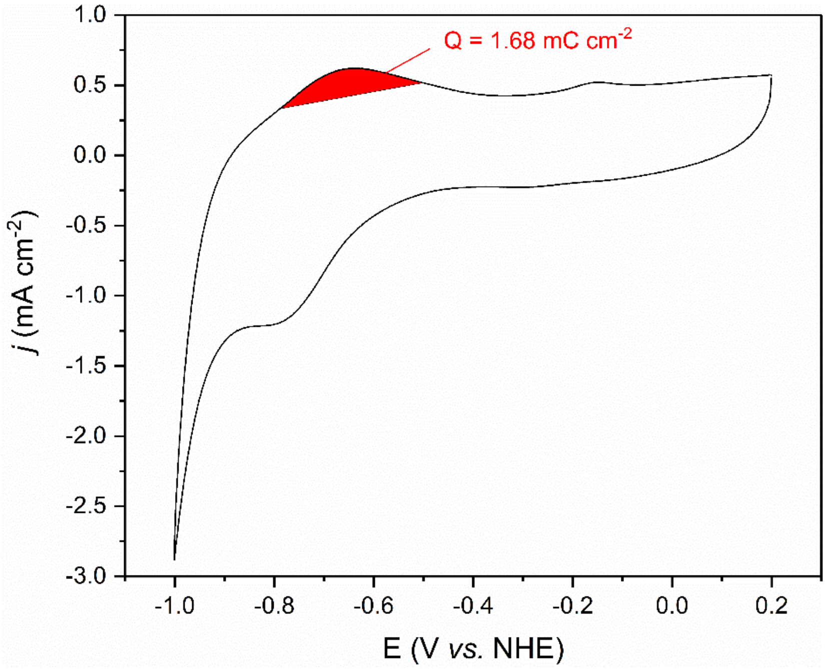

Figure S15. CV curve for CP|CNT-**CoPPc** in N_2 saturated pH 8.3 aq. HCO_3^-/CO_3^{2-} (0.5 M), ν = 20 mV s^{-1}, r.t. Electroactive cobalt was quantified using the reported method with the same catalyst.[6] The highlighted area shows the integration of the Co^I/Co^{II} anodic wave, determined using the EC-Lab software. The Γ_{Co} value reported in the manuscript is the average surface concentration calculated by integration of 10 successive CV scans at 20 mV s^{-1}. (Equation (S1) was thus applied, using this average value for *Q*, and the area of the cathode, where $A_{cathode}$ ~ 0.28 cm^2).

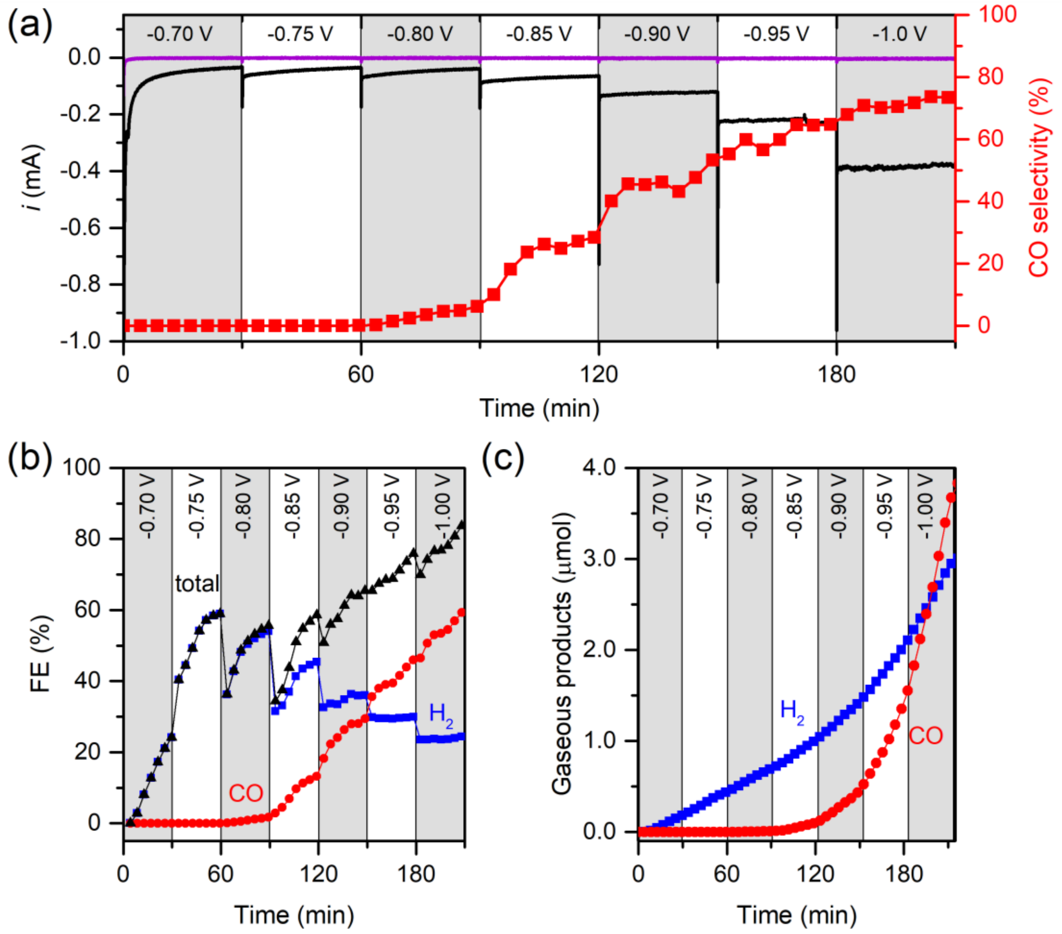

Figure S16. (a) Stepped constant potential chronoamperometry result with CP|CNT-**CoPPc** electrode (black trace) and blank CNT electrode (purple trace) under CO_2 saturated pH 7.3 aq. HCO_3^- /CO_3^{2-} (0.5 M) solution, with Pt mesh as the CE and Ag/AgCl as RE. Electrolysis was carried out in a 2-compartment cell, and the working compartment was constantly purged with CO_2 (5 sccm). Each potential step was applied for 30 min, and product formation was monitored by continuous flow GC analysis. (b) Variation in the FEs for H_2 and CO with applied potential, calculated from the total amount of gas produced during each 30 min step. *Note*: low FE observed at less negative potentials (current ~0.1 mA cm^{-2}) is likely the result of gas leakage and trapped gas bubbles in carbon nanotubes, which has a more pronounced effect on the FE at lower currents. (c) Total amount of gaseous product formed during the electrolysis experiment, with CP|CNT-**CoPPc** as the WE. No H_2 and CO were detected with the blank CNT WE.

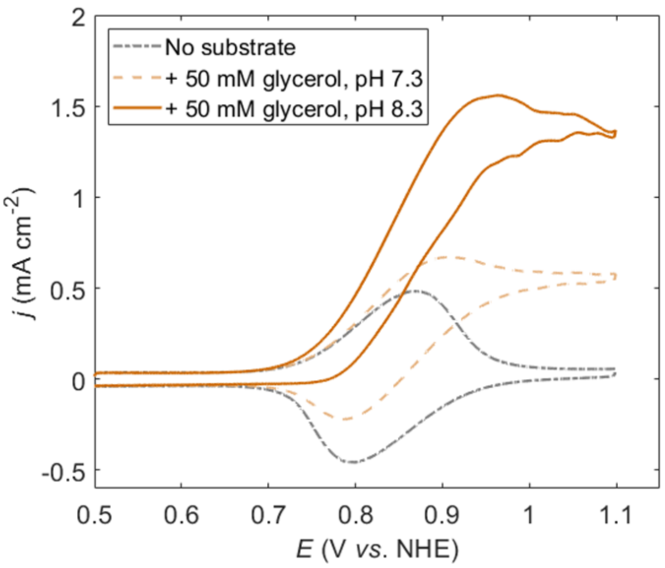

Figure S17. CV traces recorded in CO_2 saturated pH 7.3 aq. HCO_3^- /CO_3^{2-} (0.5 M) solution, and N_2 saturated pH 8.3 aq. HCO_3^- /CO_3^{2-} (0.5 M) solution, with glycerol (50 mM) as the substrate; $\nu = 20$ mV s^{-1}, r.t.

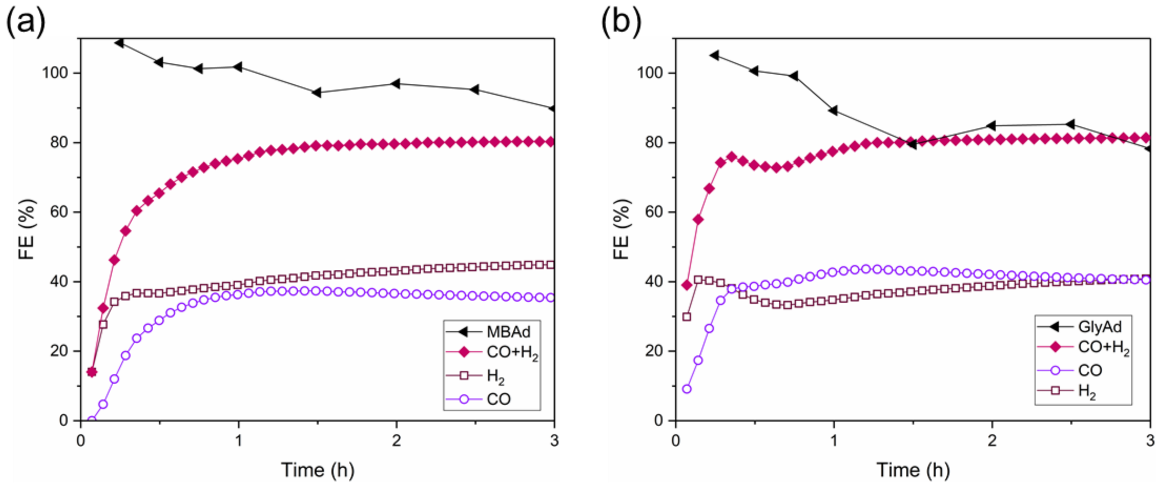

Figure S18. FEs traces over reaction time, for the oxidation and reduction products, measured for the three-electrode configuration with *meso*ITO|**STEMPO** as WE, CP|CNT-**CoPPc** as CE and Ag/AgCl as RE; (a) MBA was used as the alcohol substrate (30 mM), and (b) glycerol (50 mM).

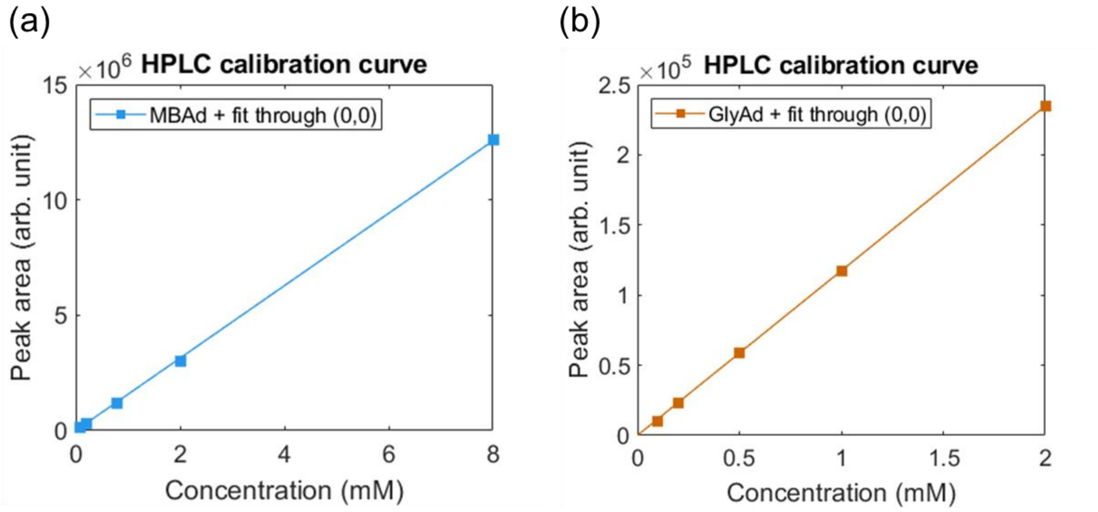

Figure S19. HPLC calibration curves for (a) MBAd, and (b) GlyAd, produced using a UV-Vis detector set at 190 nm.

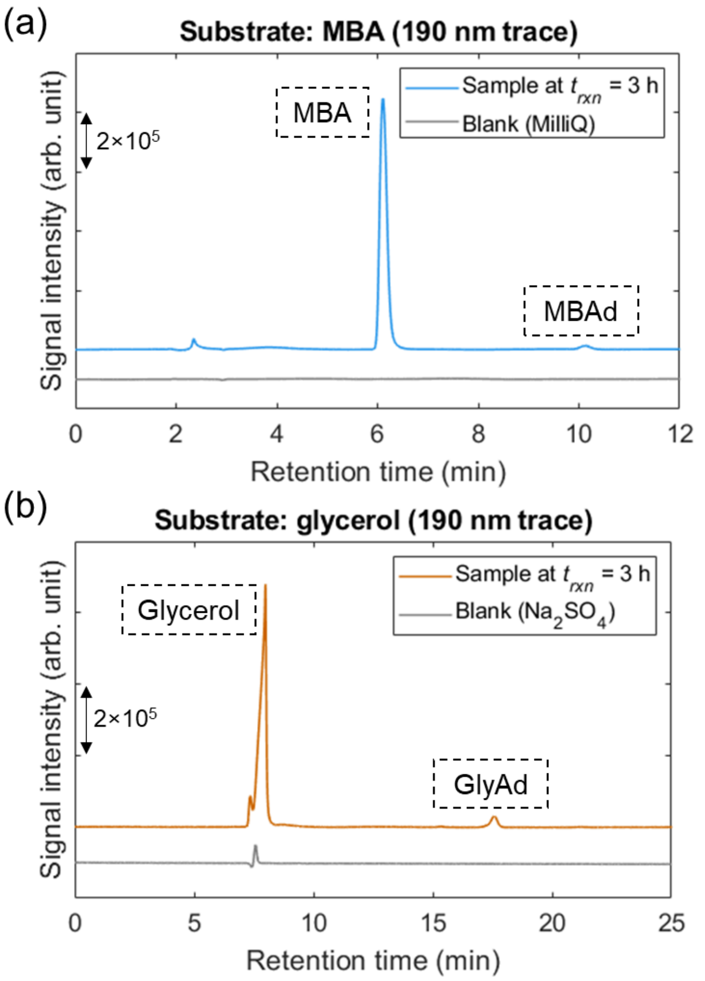

Figure S20. HPLC chromatograms (190 nm trace), corresponding to the 3 h time-point, for the coupled three-electrode CPE experiment (*meso*ITO|**STEMPO** as WE, CP|CNT-**CoPPc** as CE and Ag/AgCl as RE), invoking (a) MBA, and (b) glycerol, as the substrate. Such chromatograms were measured at regular time intervals to afford the time trace depicted in Figures 4c and 5a.

Supporting References

[1] S. G. Wan, X. Y. Yang, Y. Yu, C. Liu, *Phosphorus, Sulfur Silicon Relat. Elem.* **2005**, *180*, 2813–2821.

[2] R. Singh, J. K. Puri, V. K. Chahal, R. P. Sharma, P. Venugopalan, *J. Organomet. Chem.* **2010**, *695*, 183–188.

[3] A. M. C. Dumitriu, M. Cazacu, S. Shova, C. Turta, B. C. Simionescu, *Polyhedron* **2012**, *33*, 119–126.

[4] P. G. Hoertz, Z. Chen, C. A. Kent, T. J. Meyer, *Inorg. Chem.* **2010**, *49*, 8179–8181.

[5] M. Kato, T. Cardona, a W. Rutherford, E. Reisner, *J. Am. Chem. Soc.* **2012**, *134*, 8332–8335.

[6] N. Han, et al., *Chem* **2017**, *3*, 652–664.

[7] K. Abdiaziz, E. Salvadori, K. P. Sokol, E. Reisner, M. M. Roessler, *Chem. Commun.* **2019**, *55*, 8840–8843.

[8] S. Stoll, A. Schweiger, *J. Magn. Reson.* **2006**, *178*, 42–55.

[9] C. M. Hanna, C. D. Sanborn, S. Ardo, J. Y. Yang, *ACS Appl. Mater. Interfaces* **2018**, *10*, 13211–13217.

[10] C. Costentin, S. Drouet, M. Robert, J. M. Savéant, *J. Am. Chem. Soc.* **2012**, *134*, 11235–11242.

[11] R. P. V. Faria, C. S. M. Pereira, V. M. T. M. Silva, J. M. Loureiro, A. E. Rodrigues, *Ind. Eng. Chem. Res.* **2013**, *52*, 1538–1547.

[12] S. Verma, S. Lu, P. J. A. Kenis, *Nat. Energy* **2019**, *4*, 466–474.

[13] A. J. Bard, R. Parsons, J. Jordan, Eds., *Standard Potentials in Aqueous Solution*, New York, **1985**.

End of Supporting Information
